# Supplementary material for: Direct prediction of intrinsically disordered protein conformational properties from sequence
Source: Nat Methods. 2024 Jan 31;21(3):465–76. doi: 10.1038/s41592-023-02159-5 (PMC10927563; doi:10.1038/s41592-023-02159-5)
Supplement: Supplementary file 1 — Supplementary Methods, Supplementary Figs. 1–20 and Supplementary Tables 1–8. [file 41592_2023_2159_MOESM1_ESM.pdf]

nature methods

Article
https://doi.org/10.1038/s41592-023-02159-5

Direct prediction of intrinsically disordered protein conformational properties from sequence

In the format provided by the authors and unedited

SUPPLEMENTARY INFORMATION FOR:
Direct Prediction of Intrinsically Disordered Protein Conformational Properties From Sequence

Jeffrey M. Lotthammer*, Garrett M. Ginell*, Daniel Griffith*, Ryan J. Emenecker, Alex S. Holehouse†

Department of Biochemistry and Molecular Biophysics Washington University School of Medicine, St. Louis, MO, USA

Center for Biomolecular Condensates, Washington University in St. Louis, St. Louis, MO, USA

* These authors contributed equally
Correspondence: †alex.holehouse@wustl.edu

1. Supplementary Methods

ALBATROSS, GOOSE, SPARROW, and Metapredict V2-FF
To avoid confusion, we offer a quick summary of the specific tools discussed in this manuscript.

ALBATROSS is the set of networks used for predicting IDP ensemble dimensions directly from sequence, and the main "product" associated with this manuscript. ALBATROSS is accessible by installing sparrow (described below), and R_g and R_e predictions can also be made via our metapredict webserver (https://metapredict.net/).

Metapredict V2-FF is our updated disorder predictor, which we developed in tandem with ALBATROSS to ensure ensemble prediction and disorder prediction was on par with one another in terms of throughput.

Metapredict V2-FF provides identical predictive power to Metapredict V2 but offers a huge improvement in performance. Metapredict is available at https://github.com/idptools/metapredict, is distributed as the default version on PyPI (https://pypi.org/project/metapredict/), is accessible via our web interface (https://metapredict.net/), and as a batch Google Colab notebook (https://colab.research.google.com/drive/1UOrOxun9i23XDE8IFo_4I89Tw8P3Z1D-?usp=sharing)

SPARROW is our sequence analysis package within which the ALBATROSS networks are implemented. SPARROW (https://github.com/idptools/sparrow) offers a wide range of sequence analysis tools (some published, many not), and ALBATROSS is one of many approaches that can be performed using SPARROW.

GOOSE is our rational design tool for designing synthetic IDRs[1]. GOOSE is available at (https://github.com/idptools/goose/tree/main) with documentation at (https://goose.readthedocs.io/).

Mpipi fine-tuning

Mpipi is a one-bead-per-residue coarse-grained force field that was parameterized via a bottom-up, data-driven approach using statistics obtained from the PDB coupled with quantum mechanical calculations and all-atom simulations to derive parameters for a Wang-Frenkel (WF) potential **(Equation 1.1-1.2)** [2,3].

$$\phi(r) = \epsilon\,\alpha\left(\left[\frac{\sigma}{r}\right]^{2\mu} - 1\right)\left(\left[\frac{R}{r}\right]^{2\mu} - 1\right)^{(2\nu)}$$ **Equation 1.1**

where

$$\alpha = 2\nu\left(\frac{R}{\sigma}\right)^{2\mu} \times \left(\frac{1+2\nu}{2\nu\left[\left(\frac{R}{\sigma}\right)^{2\mu} - 1\right]}\right)^{(2\nu+1)}$$ **Equation 1.2**

Where $R = 3\sigma$ and $\nu = 1$, as defined in the original Mpipi paper.

The WF potential provides a computationally convenient (efficient to compute, intercepts with 0 at long intermolecular distances) closed-form alternative to the more commonly used Lennard-Jones potential. Given the data-driven approach used for parameterization, Mpipi benefits from explicitly encoded inter-residue interaction values (i.e., $\varepsilon_{i,j}$ values) for all unique pairs of amino acids. This is in contrast to most other one-bead-per-residue force fields, where intrinsic residue-specific interaction strengths (i.e., ε_i or λ_i) are defined, and inter-residue interaction energies are then computed via so-called 'mixing rules'.

While Mpipi offers improved flexibility for capturing chemically complex interactions, the model also has many more parameters than most conventional force fields (i.e., $[n^2 + n]/2$ interaction parameters for a model with *n* amino acids). As such, despite the excellent accuracy of the original model, we sought to determine if Mpipi could be further improved for certain sequence chemistries.

We focused on four specific groups of pairwise interactions to fine-tune Mpipi. In doing so, we developed an augmented version called Mpipi-GG. In summary, Mpipi-GG has stronger Gly:Gly and Gly:Ser interactions, weakened aromatic:charge interactions, an increased excluded volume of proline residues, and reparameterized aliphatic residues to have increased hydrophobicity (**Supplementary Figure S1**).

The adjustment to proline was motivated by the observation that upon simulation with the original Mpipi parameters, many proline-rich IDRs were too compact compared with experiments (**Supplementary Figures S2A, B**). Proline predominantly drives IDR expansion via backbone restrictions and favorable solvation[4–7]. We reasoned that tuning the proline σ parameter (i.e., its excluded volume) would enhance its expansion-driving effects. To this end, after systematically titrating potential σ values and comparing the outcome of altering the parameters with all-atom simulations (**Supplementary Figure S2C**), we increased the proline σ by 33% for all pair-wise proline interactions, as shown in **Supplementary Figure S2B**. Applying this fix improved accuracy with respect to proline-rich IDRs with minimal loss of accuracy for other IDRs (**Supplementary Figure S2D**).

Following our adjustment of proline, we examined several polar-rich homo- or dipolymeric tracts for which experimental data have previously been obtained; poly-(GS), poly-(G), poly-(S), and poly-(Q). Previous work established that sufficiently long polyglutamine (poly-(Q)) tracts form compact globules, consistent with results from Mpipi simulations[8]. However, we noticed that both poly-(GS) and poly-(G) scaled as a self-avoiding random walk (ν = ~0.60), despite the fact experimental work has suggested poly-(GS) behaves akin to a Gaussian chain (ν = ~0.5-0.55) and poly-(G) forms compact ensembles (ν = ~0.4) [9–12] (**Supplementary Figure S3A**). To address this discrepancy, we performed a titration series for poly-(G) chains. We tuned the G:G interactions by titrating the strength of the glycine-glycine attractive parameter in the WF potential ($\varepsilon_{G,G}$) for a poly-$(G)_{80}$ chain (**Supplementary Figure S3B**). Fitting these data to a coil-to-globule transition, we extracted the interaction strength (2.19x the original $\varepsilon_{G,G}$) that gave an apparent scaling exponent of 0.39, in line with previous experiments (**Supplementary Figure S3C**)[12,13]. Having established the correction factor for $\varepsilon_{G,G}$, we applied this same factor to the $\varepsilon_{G,S}$, such that poly-(GS) shows a slightly more compact scaling (ν = ~0.58) but is substantially more compact in terms of absolute dimensions, in better agreement with experiment (**Fig. 3D**). While this is not in perfect agreement with experimental work, it is an improvement on prior behavior. Without a reliable benchmark for poly-(S) we did not tune the $\varepsilon_{S,S}$ and instead focused on the $\varepsilon_{G,G}$ and $\varepsilon_{G,S}$ values. In summary, these changes significantly improve the expected polymer scaling behavior for glycine-rich sequences compared to the original Mpipi parameters.

To tune charge-aromatic interactions in Mpipi-GG we used aromatic-aromatic interactions as a benchmark. We compared the fraction of charged residues (FCR) and the fraction of aromatic residues with deviations in radii of gyration from experiment (ΔR_g). Our analysis revealed that Mpipi tended to over-compact sequences with greater aromatic and charge fractions (**Supplementary Figure S4A**). We next plotted the pairwise WF-potentials, which suggested that the arginine, aspartic acid, and glutamic acid to aromatic interaction strengths were overestimated, likely driving this compaction (**Supplementary Figure S4B**). Therefore, we tuned the $\varepsilon_{RED,FYW}$ values by systematically titrating to better fit the radii of gyration for these sequences (**Supplementary Figure 4A, right**). The final $\varepsilon_{RED,FYW}$ value was 60% lower for the

Mpipi-GG parameters. We confirmed our modifications better matched experimental radii of gyration by comparing the root mean squared error (RMSE) between simulated and experimental R_g values at different fractions of aromatic residues for the Mpipi-GG and original parameters shown in **Supplementary Figures S4C** and **S4D**

The final set of parameter modifications focuses on aliphatic residues. Aliphatic residues in the original Mpipi force field have very weak interaction strengths. To incorporate hydrophobicity, we made use of the Kyte-Doolittle hydropathy (KD_{hyro}) scale to reparameterize pairwise aliphatic interactions, such that ε_{ij} values are proportional to the sum of $KD_{hyro\ i+j}$ for a pairwise aliphatic interaction of i:j. **(Supplementary Figures S5A, B)**. Specifically, we modulated the aliphatic $\varepsilon_{AMLVI,AMLVI}$ values to strengthen aliphatic:aliphatic interactions (**Supplementary Figure S5B**). While this enhancement in hydrophobicity goes some way to improving hydrophobic interactions, we tentatively suggest hydrophobicity is still broadly underestimated in Mpipi-GG; as such, we suggest caution when interpreting results associated with sequences enriched for aliphatic residues.

In summary, small changes were made to parameters associated with twelve of the twenty natural amino acids: proline, glycine, arginine, aspartic acid, glutamic acid, phenylalanine, tyrosine, tryptophan, alanine, valine, isoleucine, leucine, and methionine. All changes made to the interaction matrix can be visualized in **Supplementary Figure S1**, which reports on the change in the overall interaction parameter between Mpipi-GG and Mpipi. We also provide a complete parameter file for Mpipi-GG at the main GitHub repository associated with this paper under (at `simulations/lammps_mpipi_ggv23.in`) which includes comments for each parameter line that has been edited.

The overall interaction parameter reports the net integral of the short-range (Wang-Frenkel) and long-range (Coulombic) interaction potentials. We emphasize that these changes were made explicitly with single-chain behavior in mind and have not been tested regarding their impact on phase behavior. As such, while the original Mpipi model may be preferable for studying two-phase systems, we proceeded to use Mpipi-GG for single-chain sequence-ensemble predictions.

SAXS data used for comparison

We assessed the accuracy of Mpipi using extant SAXS data obtained from the literature, as described in previous work[14]. Wherever possible, we re-analyzed primary scattering data to ensure that reported values matched the radii of gyration reported in prior publications, although we always report the R_g as previously published. The complete set of sequences and all Rg values (from all models tested and SAXS data) are provided as supplementary information (**Supplementary Table S8**).

IDR sequence library design

To construct *bona fide* disordered protein sequences, we leveraged the software package GOOSE, which enabled us to construct libraries of rationally designed disordered proteins with specific yet broad sequence chemistries[1]. Therefore, we first designed disordered sequences with varying Fractions of Charged Residues (FCR), Net Charge per Residue (NCPR), and Kyte-Doolittle hydropathy scale values. In addition, we also generated disordered sequences with randomly assigned but specific amino acid fractions (where remaining amino acids were unrestrained) to sample across the sequence space accessible to disordered regions. Finally, we also ensured that we had broad coverage of charge distribution in our generated sequences by titrating across kappa, a charge asymmetry parameter where higher values mean greater charge asymmetry.

All-atom Excluded Volume (EV) simulations

Coarse-grained excluded volume (EV) simulations were performed in Mpipi-GG by adjusting the epsilon and sigma parameters such that the interaction potential overlaps for the repulsive component of the function but flattens to zero for distances greater than σ (**Supplementary Figure S16**). All-atom EV simulations for polyproline (**Supplementary Figure S2**) were performed using the CAMPARI simulation engine (V2) https://campari.sourceforge.net/) and the ABSINTH implicit solvent model[15]. EV simulations were performed as done previously[4,16]. Briefly, EV simulations involve scaling the attractive Lennard-Jones component, the solvation component, and the electrostatic component of the ABSINTH Hamiltonian to zero, such that the only determinant of the underlying ensemble reflects the excluded volume dictated by the repulsive component of the Lennard-Jones potential.

Scaled network training

For both the radius of gyration and the end-to-end distance networks, we trained BRNN-LSTM networks with and without sequence length normalization. For the normalized variations, we performed normalization by taking the respective metric and dividing it by the square root of the sequence length. The radius of gyration (or analogously the end-to-end distance) for a polymer can be defined as $R = A_0 \times N^{\nu}$ where R is either the radius of gyration or end-to-end distance, N is the length of the sequence, and $\boldsymbol{\nu}$ is the scaling exponent. A Gaussian chain is a chain that scales with $\boldsymbol{\nu}$ as 0.5. Therefore, to obtain the length-independent (i.e., sequence chemistry) contribution to the chain dimensions, one can normalize R by the root of the sequence length to derive the following relationship $\frac{R}{\sqrt{(N)}} = A_0 \times N^{(\nu - 0.5)}$. This scaling normalizes the measure in polymer space and standardizes the ensemble dimension such that length is a less dominant factor of the learned network. For each scaled network, we followed the same 5-fold cross-validation procedure for hyperparameter tuning, and the final network weights were selected from the lowest validation loss across 750 epochs.

ALBATROSS distribution

In addition to providing a locally installable implementation of ALBATROSS via SPARROW, we also created a point-and-click style interface for ALBATROSS hosted on Google Colab to eliminate the software barrier of entry for users.

By leveraging the cloud computing resources provided in Google Colab, we enable the accurate prediction of IDR conformational properties from sequence from anywhere in the world with an internet connection - even a smart device. Moreover, our Google Colab implementation enables users to specify a single sequence or upload a fasta file of disordered protein sequences for ALBATROSS predictions. This means users can leverage the unique throughput of ALBATROSS predictions without even needing to write code to construct complex bioinformatic pipelines. Additionally, all predictions are filterable by numerical ranges for that property. As a final note, GOOSE enables the ability to design sequences with specified ALBATROSS-based ensemble predictions.

In addition to being distributed through Google Colab, the ALBATROSS networks are also integrated within the SPARROW sequence analysis package under the “predictors” object operator. In this context, proteome-scale predictions can be achieved in a few lines of code on commodity hardware, e.g.:

```
from sparrow import Protein

# create a Protein object
P  = Protein(‘MEEPQSDPSVEPPLSQETFSDLWKLLPENNVLSPLPSQAMDDLMLSPDDIEQWFTEDPGPDEAPRMPEAAPPVAPAPAAPTPAAPA’)

# predict Rg
rg = P.predictor.radius_of_gyration()

print(rg)

> 32.381092
```

ALBATROSS predictions in SPARROW are, by default, memoized such that computations are not repeated after each call to the predictor operator. An optional override is provided to recompute predictions if this is desired. The lightweight and object-oriented nature of SPARROW makes it possible to build complex bioinformatic pipelines, integrating both bioinformatic sequence properties and the emergent biophysical properties of a sequence.

In addition to performing prediction in series via SPARROW protein objects, users can perform batch predictions on both CPUs and GPUs. If GPUs are available, predictions can be obtained at 1000s of sequence predictions per second. However, even on CPUs, batch prediction offers 10-50x improvement in throughput with no loss of accuracy. For example, R_g values for all 29,998 IDRs defined by Tesei and Trolle *et al.* can be predicted in 23 seconds on a CPU using batch mode[17].

Scaled vs. unscaled networks in ALBATROSS

While scaled and unscaled networks were trained for end-to-end distance and radius of gyration (see above), scaled networks performed better across the board, especially for short sequences. Notably, unscaled networks often predicted unphysical dimensions for sequences less than 30-35 residues in length, while scaled networks performed uniformly well (**Supplementary Figures. S17, S18**). Given the better performance of the scaled networks, the default networks implemented for sparrow predictions are the scaled networks.

For sequences shorter than 35 residues, even if unscaled networks are requested, sparrow falls back to force the scaled networks to be used. This behavior can be overridden by setting `safe=False` as a parameter when performing predictions, although we do not recommend this.

ALBATROSS comparison with expectations from the AFRC

The Analytical Flory Random Coil (AFRC) – a Gaussian-chain-like model for disordered proteins – was compared to ALBATROSS to illustrate the breadth of chemistries where the gaussian-chain-like assumptions begin to break down[14]. For biological sequences, the *correlation* between the AFRC and ALBATROSS-derived predictions is extremely good, demonstrating that a substantial fraction of the predictive power in absolute value comes from the degree of polymerization (i.e. number of amino acids) (**Supplementary Figure. S19A**). That said, the absolute value is less well-correlated, with the AFRC being more compact than most naturally occurring IDRs.

In contrast, the derived radii of gyration from AFRC and predicted radii of gyration from ALBATROSS deviate substantial for sequences with diverse sequence chemistries or patternings (R^2 = 0.676, **Supplementary Figure. S19B**). The impact of sequence chemistry is more pronounced on the end-to-end distance than the radius of gyration, as illustrated by the weaker correlation between the ALBATROSS-predicted R_e and the AFRC-derived R_e (R^2 = 0.470, **Fig. S19D**).

Notable errors in ALBATROSS

Interestingly, ALBATROSS performs less accurately on short synthetic sequences with large alanine sequence fractions (**Supplementary Figures S9C, D**). We note, however, that these alanine-rich sequences are not expected to behave as *bona fide* disordered proteins and

instead adopt substantial alpha helicity. A similar trend was observed for the end-to-end distance network. Namely, shorter sequences with large alanine sequence fractions were more poorly predicted. Moreover, these challenges were largely mitigated via the scaled network training procedure. The utilization of the scaled networks for radii of gyration and end-to-end distance computations is thus the default setting, although we present an optional override for the use of the unscaled networks.

Comparison with CALVADOS2 radii of gyration

Predictions for 29,998 IDRs calculated using the CALVADOS2 force field were obtained from Tesei & Trolle et al. [17], using the .csv file obtained from https://github.com/KULL-Centre/_2023_Tesei_IDRome/tree/main. We find that CALVADOS2 and ALBATROSS predictions correlated with an R^2 of 0.98 across the human proteome (**Supplementary Figure. S15**) or 0.97 for the R_g values measured by SAXS (**Supplementary Figure S8)**. We also used the Google Colab notebook for CALVADOS2 simulations available at https://colab.research.google.com/github/KULL-Centre/EnsembleLab/blob/main/IDRLab.ipynb for our curated dataset of experimental radii of gyration from the literature.

2. Supplementary Figures

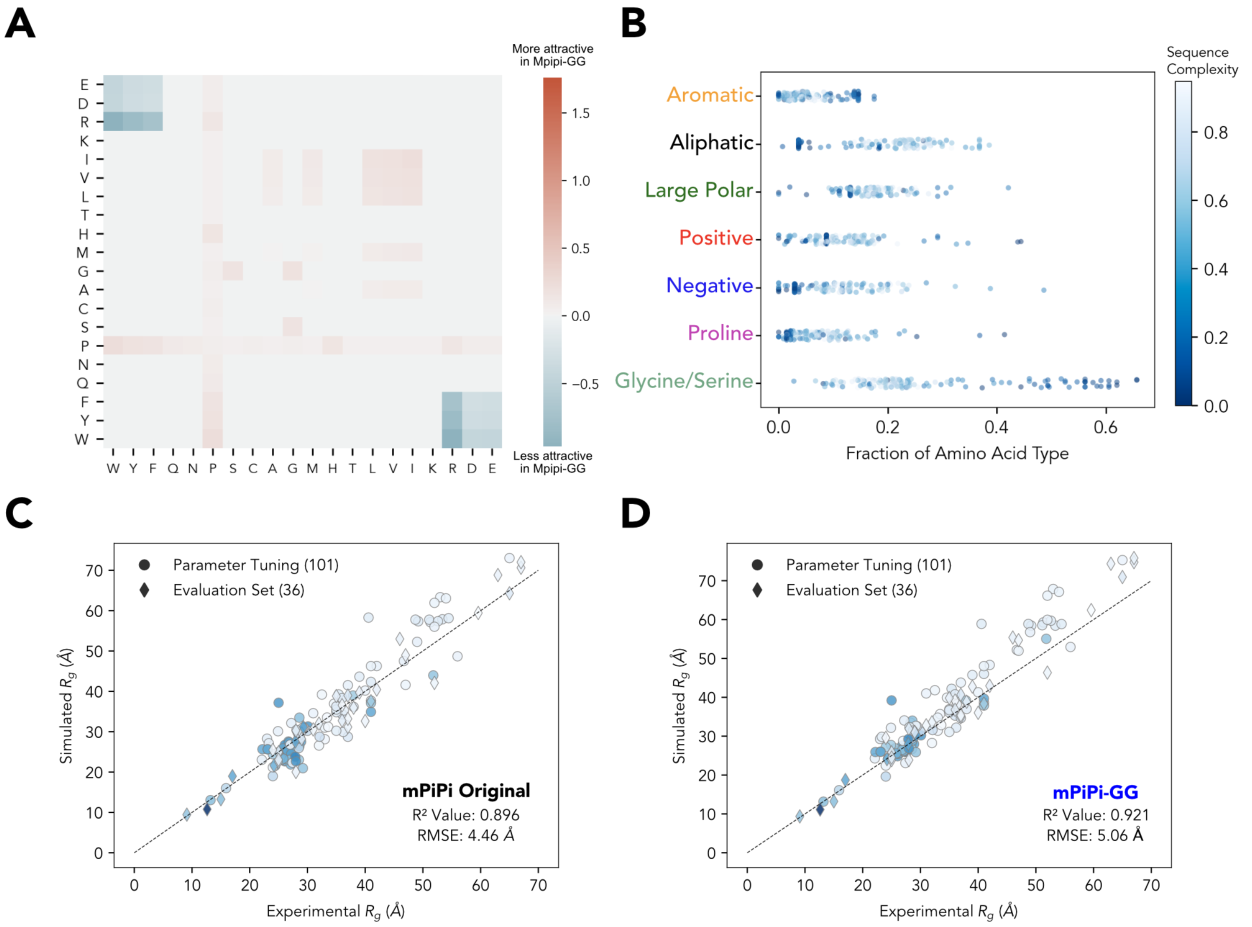

Fig. S1. Reparameterization and accuracy of the Mpipi-GG force field. A) Pairwise interaction matrix for the reparameterized Mpipi-GG force field. Pairwise interactions are colored by the relative change in interaction energies between the Mpipi force field and the new Mpipi-GG force field. Interaction energies are the sum of the net contributions from both the pairwise Coulombic interactions as well as the pairwise WF interactions. **B)** Composition of the curated experimental SAXS sequence dataset by amino acid type. The blue color gradient signifies the Wootton-Federhen complexity of the sequence. **C-D)** Correlations and RMSEs between the original Mpipi and Mpipi-GG force fields and a curated set of 137 experimental radii of gyration. 101 sequences (circles) were used for validating the Mpipi-GG force field, and 36 were new sequences held out during parameter fitting (diamonds). The same color scale used in B is used in panels C and D.

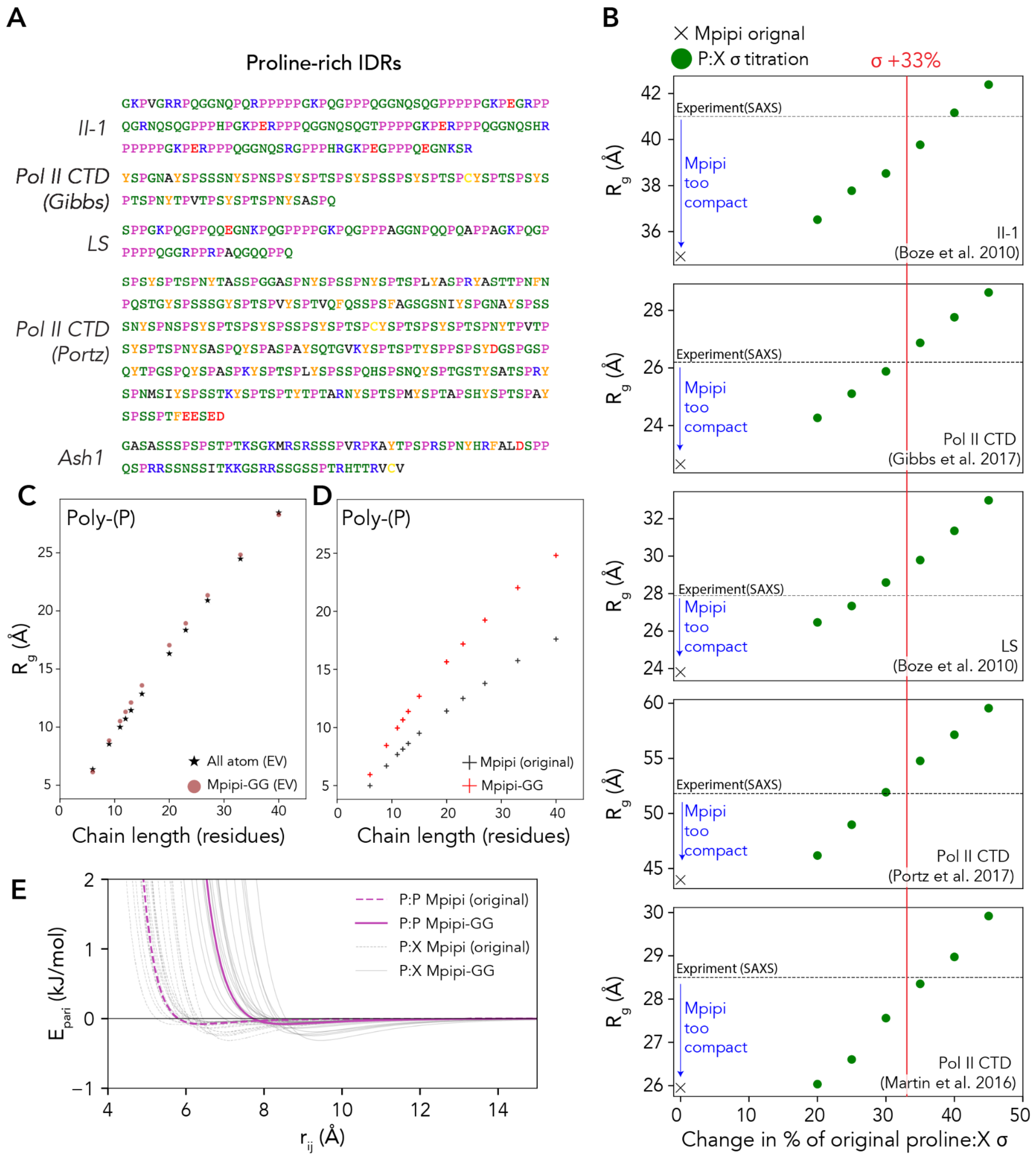

Figure S2. Tuning of proline sigma (σ) parameter in the Mpipi force field

A) Amino acid sequence of proline-rich IDRs previously studied by small angle X-ray scattering (SAXS)[4,6,18,19]. **B)** Comparison of experimental (dashed line) with predicted R_g obtained from Mpipi original ('X' tick, far left) demonstrates that proline-rich IDRs are overly compact in Mpipi. This compaction can be alleviated by increasing the σ parameter from the WF potential (see equation 1), in effect, making proline residues larger. Green points represent the result of a systematic titration of the σ value. **C)** The optimal change to the proline σ parameters was selected by comparing excluded volume (EV) coarse-grained simulations from Mpipi with all-atom EV simulations and identifying the σ value that results in consistent R_g vs. N scaling.

Shown here is a comparison of a +33% increase (used in Mpipi-GG) for Mpipi-GG EV simulations vs. all-atom EV simulations. **D)** Final comparison of polyproline dimensions for Mpipi-GG vs. original Mpipi. Mpipi-GG is more expanded than the original Mpipi, as also shown by the better agreement with experiment at a 33% increase, as shown in panel B. **E)** Wang-Frenkel (WF) potential for Pro:Pro interaction in the original Mpipi parameters (dashed purple line) vs. Mpipi-GG (solid purple line). Dashed and solid gray lines represent proline and each of the other twenty amino acids for Mpipi and Mpipi-GG, respectively.

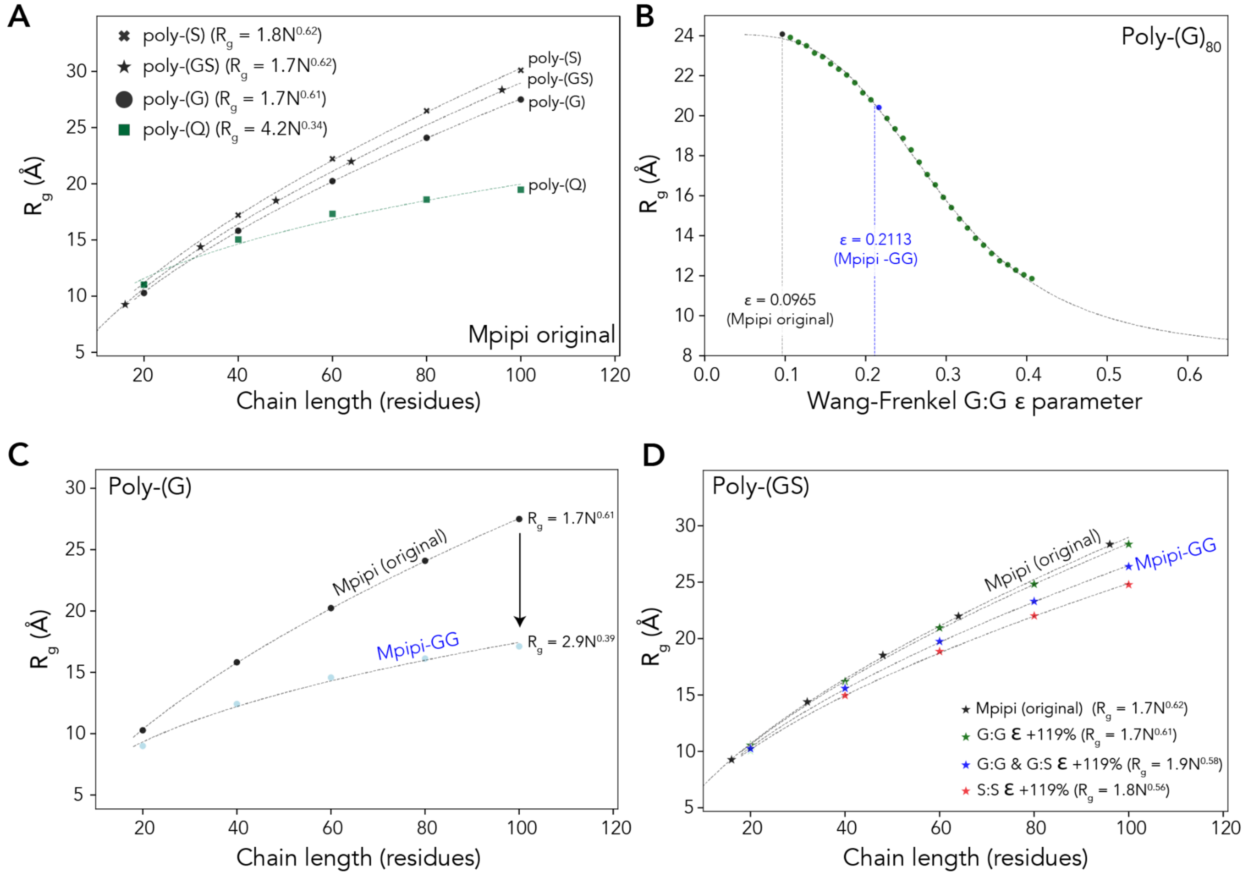

Figure S3. Gly/Ser Mpipi-GG reparameterization A) Simulated scaling behavior for simple polymeric sequences performed using the original Mpipi model. Poly-(Q) compaction is consistent with experimental work[8]. However, poly-(G) scales as a self-avoiding random chain, despite prior work implicating a scaling exponent closer to 0.40[12]. Further, poly-(GS) scales as a self-avoiding random walk (ν = 0.6) against prior work from simulations and experiments, which suggest poly-(GS) sequences behave closer to a Gaussian chain (ν = 0.5 - 0.55) [8–11]. These data suggest that G:G interactions are too weak. **B)** To reparameterize G:G strength we systematically titrated the glycine ε parameter, leading to a coil-to-globule titration from which we selected the ε value that best matches the expected scaling of 0.4. **C)** Comparison of Mpipi vs. Mpipi-GG, revealing the more compact scaling and smaller scaling exponent (0.39 vs. 0.61), in better agreement with experiment. **D)** Despite strengthening G:G interactions, poly-(GS) dipeptide repeat polymers are still relatively expanded (compare black and green data). To address this, we asked how changing the S:S: interaction (red) vs. G:G and G:S (blue) altered

chain dimensions. Given the prevalence of serine residues in disordered regions, we released the Mpipi S:S interaction strength was likely already reasonable, such that we selected the same scaling for G:G and G:S to enhance cohesive interactions between glycine and serine.

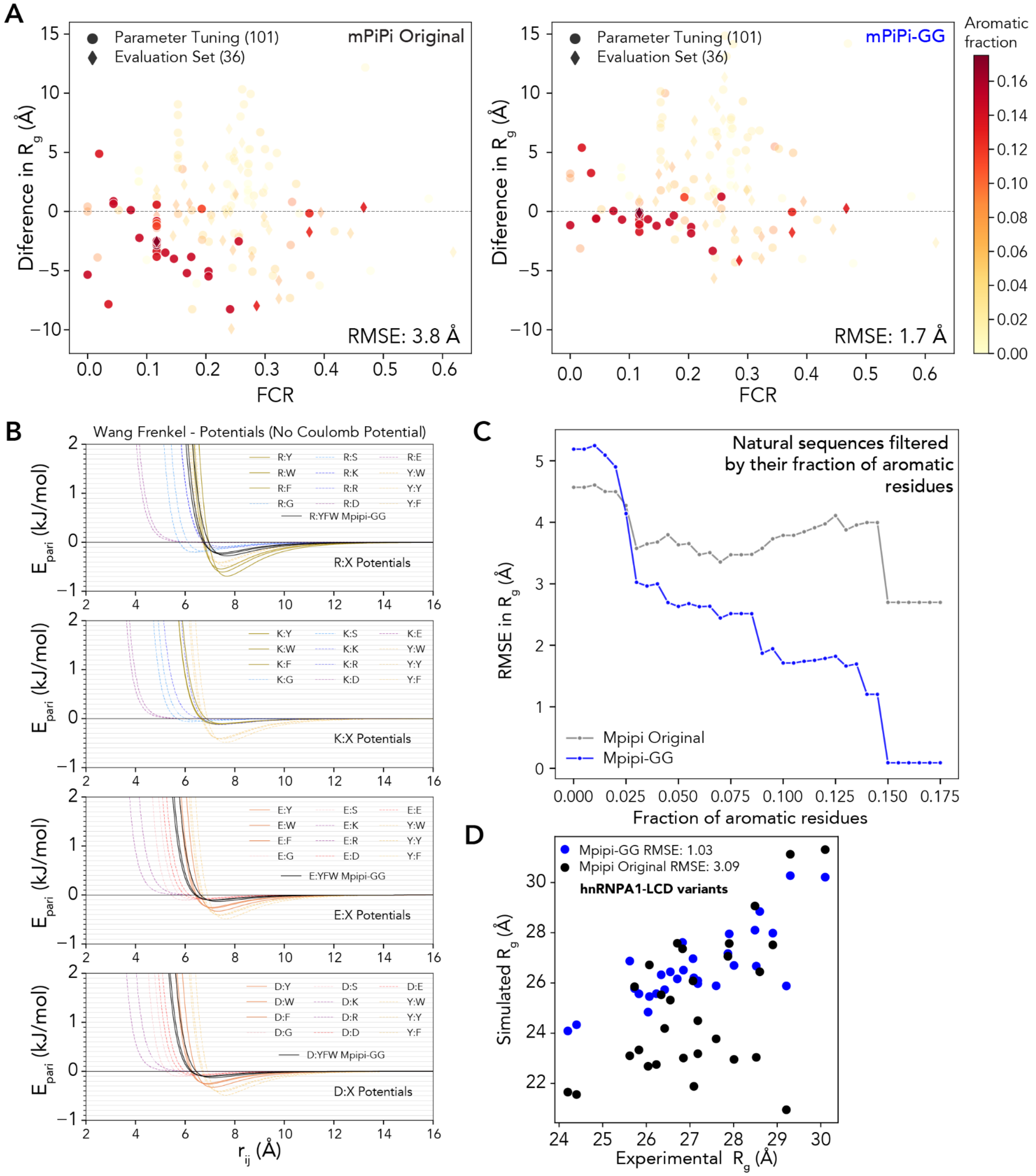

Figure S4. Reparameterization of pairwise aromatic interactions for the Mpipi-GG force field. A) Residual plot for the deviations between experiment and Mpipi (left) and Mpipi-GG (right) as a function of the fraction of charged residues. When comparing all sequences in our curated dataset with >10% aromatic residues, the original Mpipi has an RMSE of 3.8 Å, whereas Mpipi-GG has an RMSE of 1.7 Å. We note specific improvement in sequences that are jointly aromatic and charge rich - i.e., >10% of charged residues by fraction. **B)** Pairwise Wang-Frenkel interaction potentials for arginine, lysine, aspartic acid, and glutamic acid relative to aromatic residues (solid lines) and benchmark residues (dotted lines). Updated Mpipi-GG

potentials are drawn in black. **C)** Panel **A** uses an aromatic threshold value of 0.10; however, this choice is somewhat arbitrary. Therefore, we demonstrated generality by looking at many different potential thresholds. This plot looks at the root mean squared error as a function of aromatic thresholding. For each threshold value (x-axis), we took all sequences in the curated dataset with aromatic amino acid fractions equal to or exceeding the respective threshold value and computed the RMSE between the experiment and simulated results for each respective force field. As aromatics fractions are increased, Mpipi-GG consistently has modest improvements in recapitulating experimental SAXS radii of gyration. RMSEs near zero in Mpipi-GG are reflective of the fact that there are few sequences with greater than 15% aromatics in the curated library. Nevertheless, Mpipi-GG is highly accurate for these aromatic and charge-rich sequences. **D)** Comparison of simulated R_g vs. SAXS-derived R_g for hnRNPA1-LCD variants[20,21]. These sequences systematically vary charge and aromatic content, providing a convenient reference set for comparing Mpipi-GG vs. Mpipi in the context of aromatic/charge interactions.

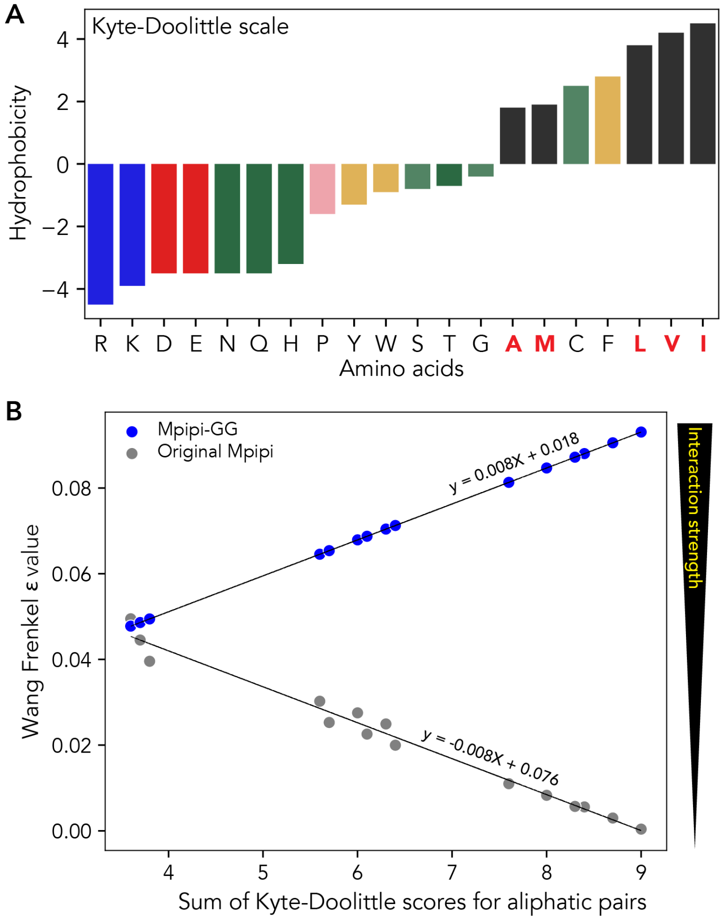

Figure S5. Reparameterization of pairwise aromatic interactions for the Mpipi-GG force field. A) Kyte-Doolittle hydropathy scale for each of the twenty amino acids. **B)** Pairwise sum of Kyte Doolittle hydrophobicity (KD_{hyro}) values relative to Mpipi $\varepsilon_{AMLVI,AMLVI}$ values. Reparameterized $\varepsilon_{AMLVI,AMLVI}$ values in Mpipi-GG, $\varepsilon_{i,j}$ are equal to 0.0008*$KD_{hyro(i+j)}$ +0.018, where the slope of this line is inversely proportional to that of the $\varepsilon_{AMLVI,AMLVI}$ values in the original Mpipi force field, but scaled so the more hydrophobic pairs are stronger, as opposed to weaker.

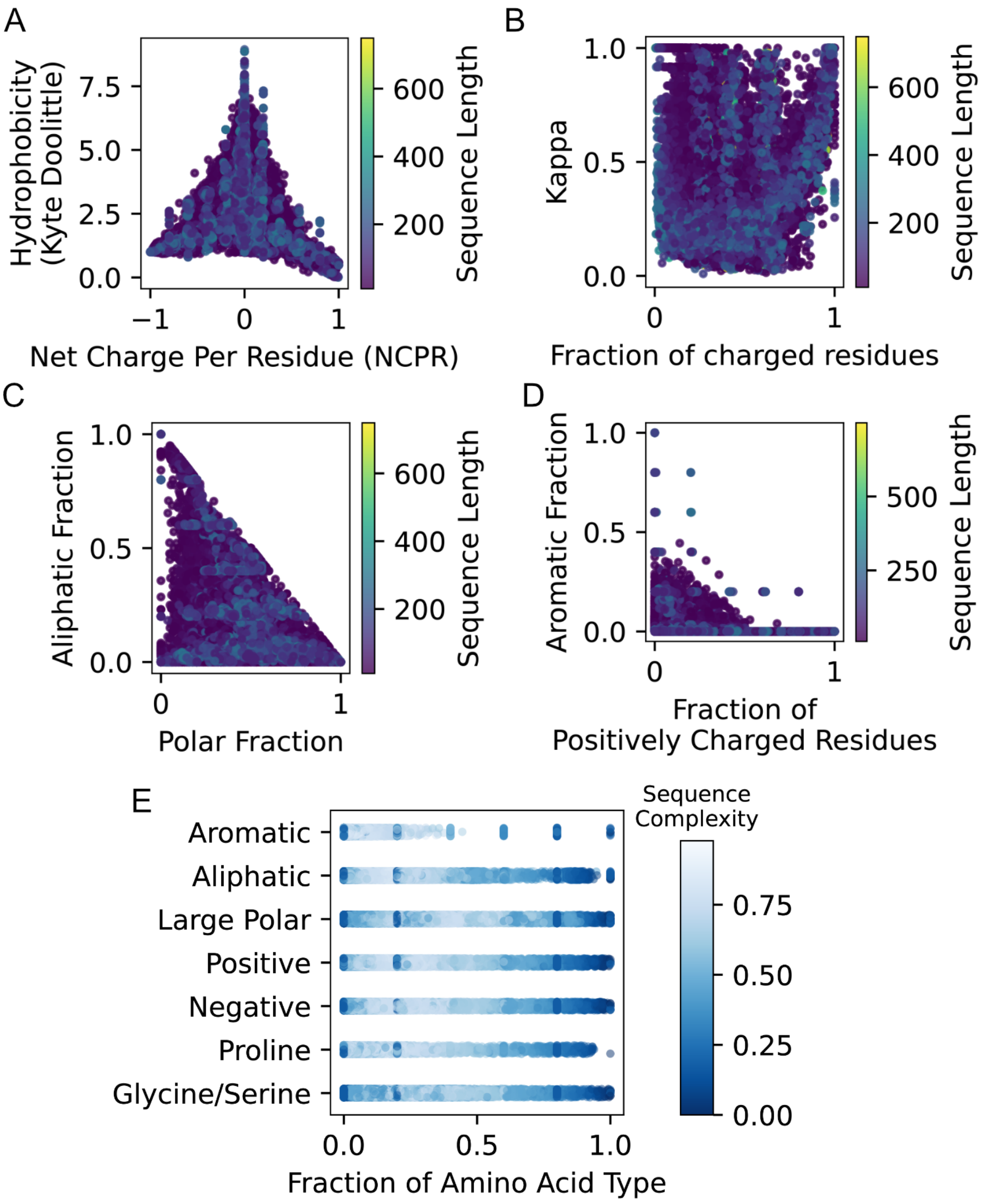

Fig. S6. Composition of the IDR sequence library used for training. A) Composition of the synthetic sequence library by amino acid type. The blue color gradient signifies the Wootton-Federhen complexity of the sequence. Two-dimensional scatter plots showing the chemical space explored by our synthetic IDR library. Each point in all panels is colored by the length of that particular sequence. **B)** Fraction of polar residues versus the fraction of aliphatic residues in a given sequence. **C)** Fraction of aromatic residues and the fraction of positively charged residues (RK). **D)** Fraction of charged residues versus the charge patterning parameter kappa (κ). **E)** Distribution of sequence complexity vs. composition.

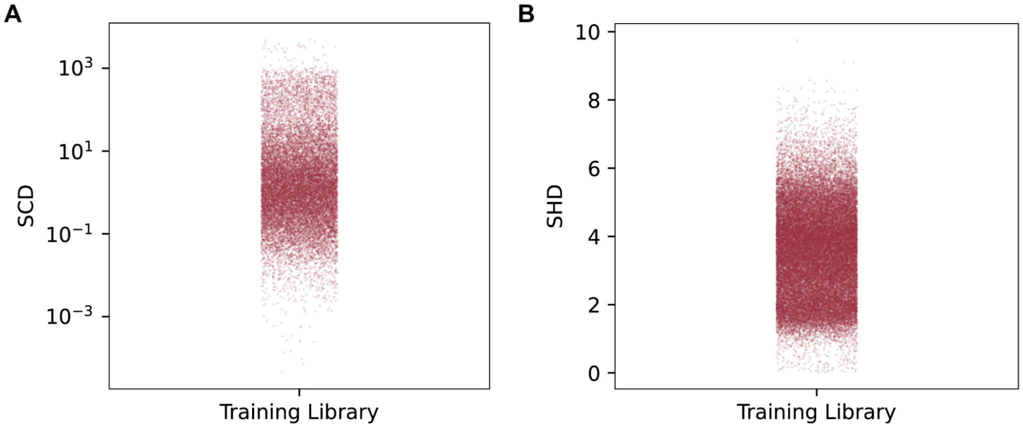

Figure S7. Sequence property comparison for the biological and synthetic sequence libraries. A) Distribution of sequence charge decoration (SCD) values for the training set of sequences. Note the SCD is plotted on a logarithmic scale as the training data covers a broad dynamic range of SCD values. **B)** Distribution of sequence hydropathy decoration values for the training set of sequences.

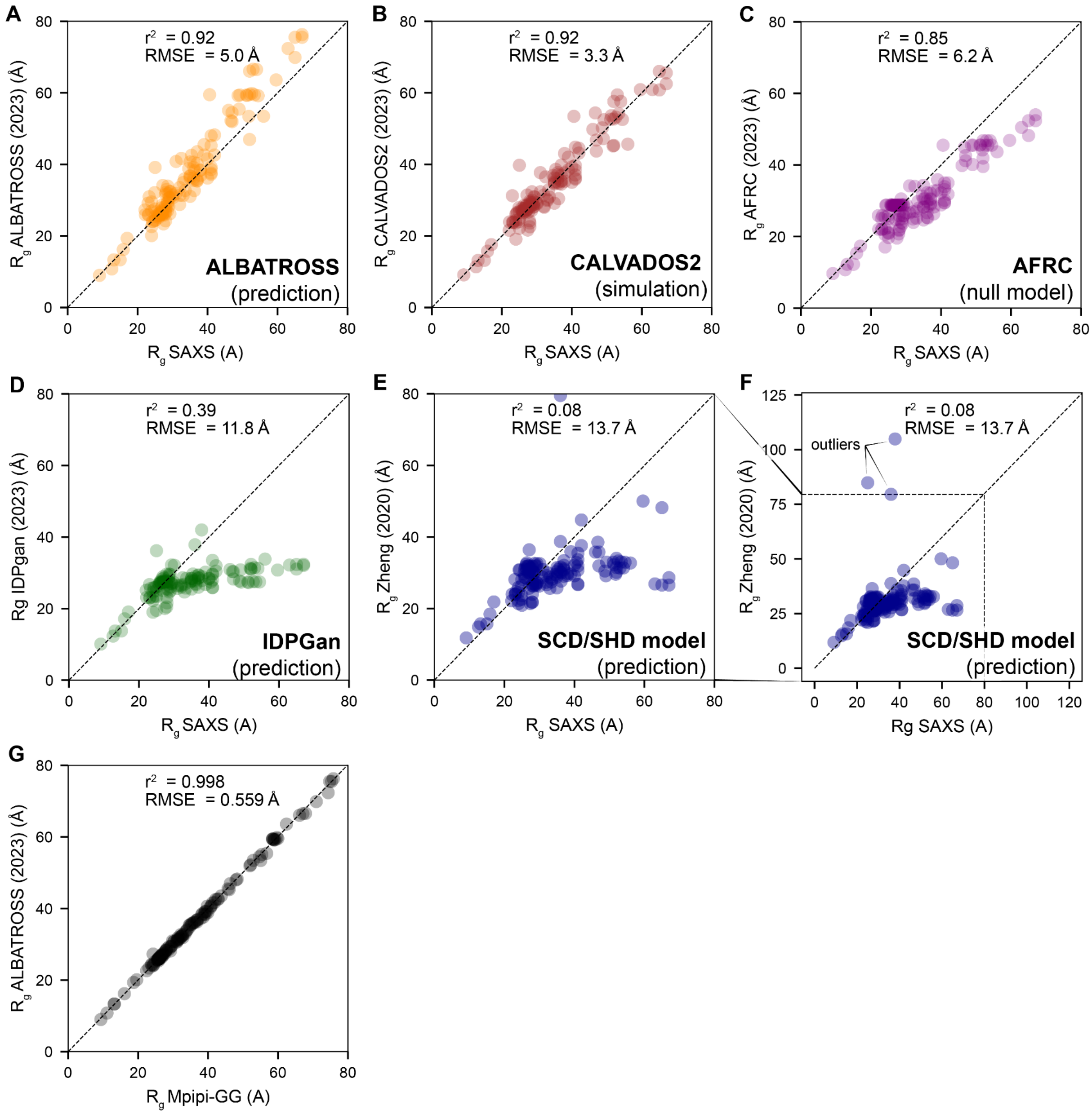

Figure S8. Comparing predictions vs. SAXS measurements for state-of-the-art tools for mapping between sequence and ensemble. A) The ALBATROSS R_g network recovers a correlation coefficient of 0.92 (RMSE = 5.0 Å), in agreement with analogous analysis for Mpipi-GG. Predicting these values in series takes ~4 seconds on a CPU. **B)** Simulations performed using CALVADOS2 show an equivalent correlation coefficient with a smaller RMSE value (RMSE = 3.3 Å)[17]. Simulations performed using the Google Colab notebook took ~ 7 minutes on GPU for a 137 residue sequence. **C)** The AFRC is an analytical model that reports the expected dimensions of a polypeptide if it behaves as a Gaussian chain[14]. Calculating anticipated R_g values for these sequences takes < 1 second for all sequences. **D)** IDPGan is a deep learning model trained to predict IDP ensembles from sequence[22]. Predictions were performed using the Colab notebook and took 2-5 seconds per sequence. **E)** The SCH/SHD

model uses a set of empirical equations derived by Zheng *et al.* to predict the radius of gyration from sequence via a predicted scaling exponent[23]. Predictions take < 1 second for all sequences. We note that the r^2 value here is strongly influenced by the presence of three major outliers (the highly-charged sequences Fez1, Histone H1-CTD, and Prothymoysin alpha). If those points are removed, the r^2 is comparable to IDPGan (r^2 = 0.32, RMSE = 10.9 Å). **F)** Same data as in panel E but with an extended x-axis and y-axis limits, highlighting extreme outliers that give rise to the noticeably poor r^2 value highlighted. **G)** ALBATROSS reproduces simulation-derived radii of gyration with almost no appreciable error.

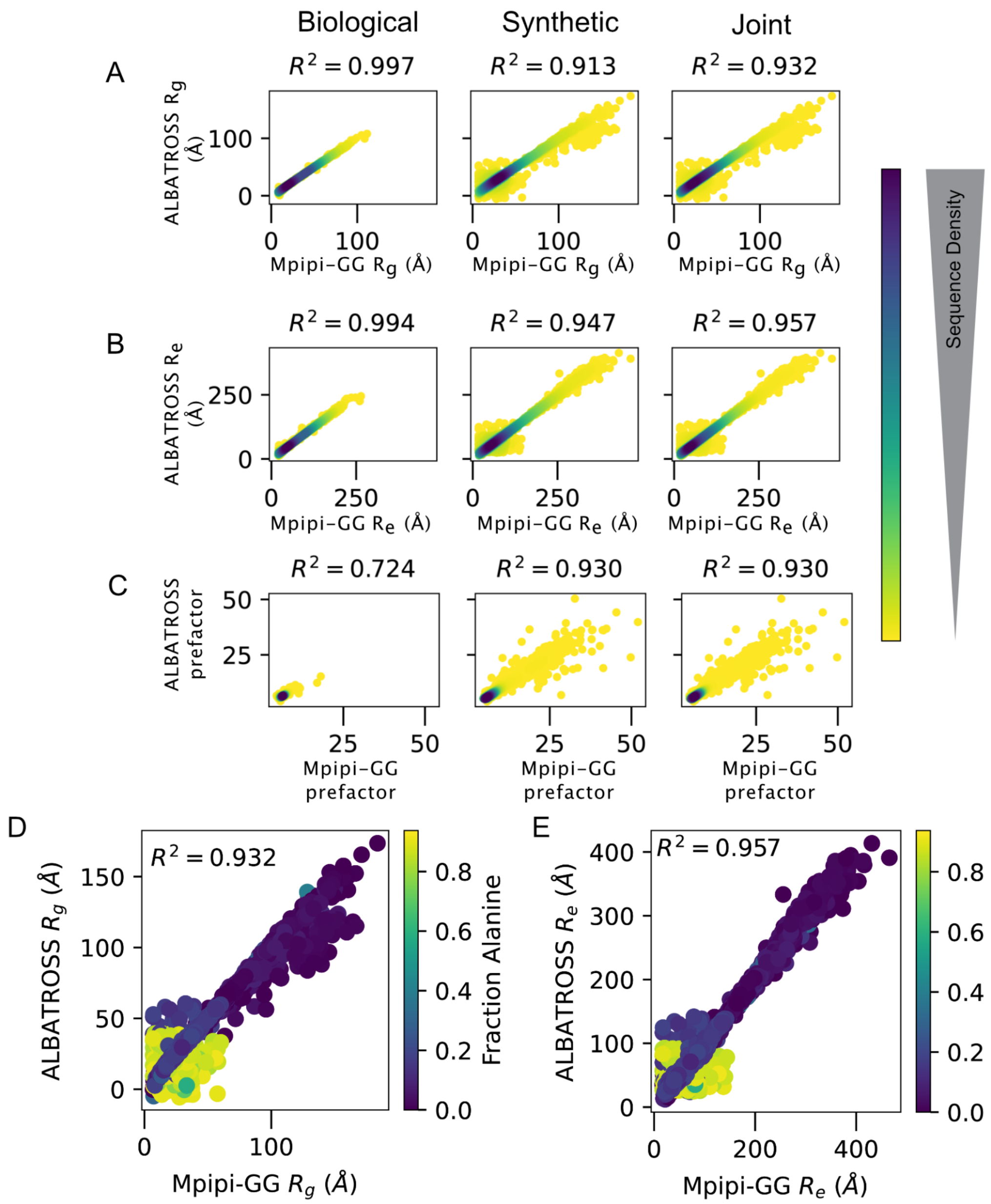

Figure S9. Evaluating the performance of the ALBATROSS networks on held-out test sets A-C) Performance of the unscaled radius of gyration network, unscaled end-to-end distance network, and polymeric prefactor for biological, synthetic, and joint test set. **D-E)** Examining the unscaled radius of gyration and end-to-end distance ALBATROSS networks. We note that for the unscaled networks, shorter sequences with large alanine sequence fractions display predictions with considerable variations from the actual simulated Mpipi-GG values. For this reason, we default to using the scaled networks trained as presented in **Fig. 2.** The alanine sequence fraction colors data points.

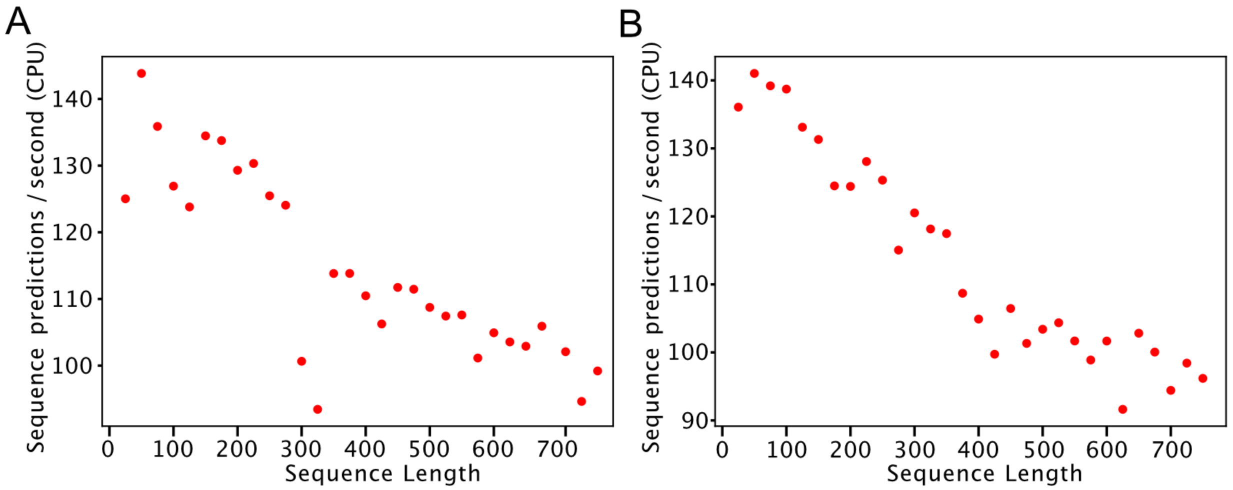

Figure S10. Network performance on standard commodity hardware. We measured predictive power on an Intel(R) Core(TM) i9-9900 CPU for **A)** the radii of gyration network and **B)** the end-to-end distance network as a function of sequence length. For 100-residue IDRs, performance sits around 120-140 sequences per second. We emphasize that on a Google Colab notebook using GPUs, predicting the IDRs and their corresponding ensemble properties for the entire human proteome takes ~8 seconds. However, we focussed our benchmarking on CPU performance given their broad availability. Comparable performance (100s of sequence predictions per second for 100-residue IDRs) is obtainable using both the Intel and M1 Macbook CPU cores. We provide a benchmarking notebook in our supporting data repository available at: https://github.com/holehouse-lab/supportingdata/tree/master/2023/ALBATROSS_2023/manuscript/si_figures/s10.

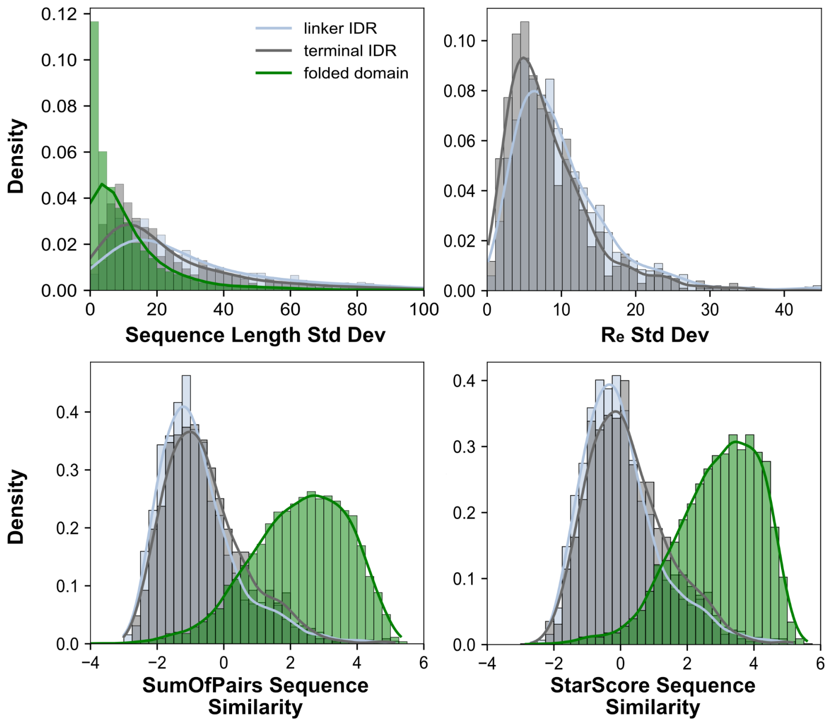

Figure S11. Distribution of predicted R_e standard deviation and sequence similarity standard deviations across yeast homologs. Histograms describe the scatterplot data presented in **Fig. 6B** and **S12**. The SumOfPairs sequence similarity metric was computed with pyMSA. The StarScore method is another approach for computing the similarity between sequences that was leveraged to test for sensitivity to the chosen similarity metric. In both cases, negative values correspond to more divergent primary sequences. For more information, see the Yeast homologous IDR Analysis section in the *Methods*. The distribution of homologous folded domains for sequence similarity metrics is plotted for comparison. As expected, folded domains tend to show much greater sequence similarity than disordered regions.

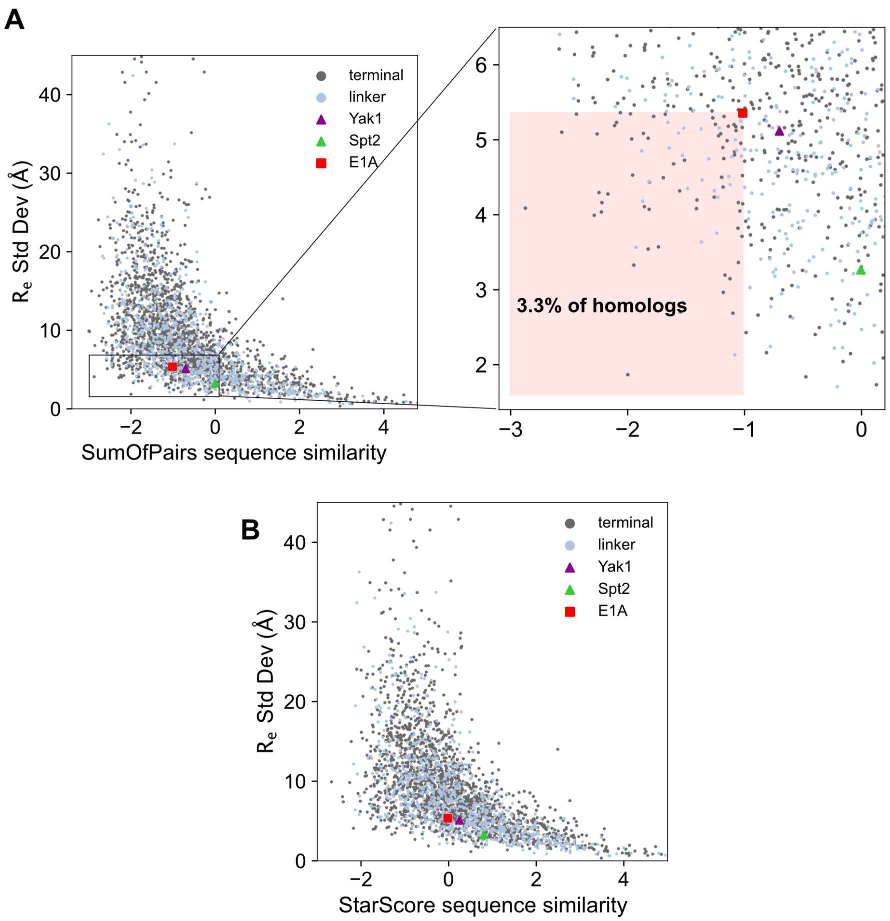

Figure S12. Multiple sequence alignment-based sequence similarity analysis of yeast IDR homologs. A) Scatterplot to complement main text **Fig. 6B** by depicting the sequence similarity between homologous sequences versus the end-to-end distance. The sequence similarity is computed with pyMSA using the SumOfPairs method, as described above. Negative values correspond to more divergent primary sequences. There is a trend that more divergent homologs tend to have a greater variation in predicted R_e. The panel on the right shows a zoomed-in inset of the region of the plot corresponding to homologs that have more divergent sequences and more constrained R_e than E1A. **B)** Same plot as **A**, but with sequence similarity calculated using the StarScore method, indicating this analysis is robust to the choice of specific metric.

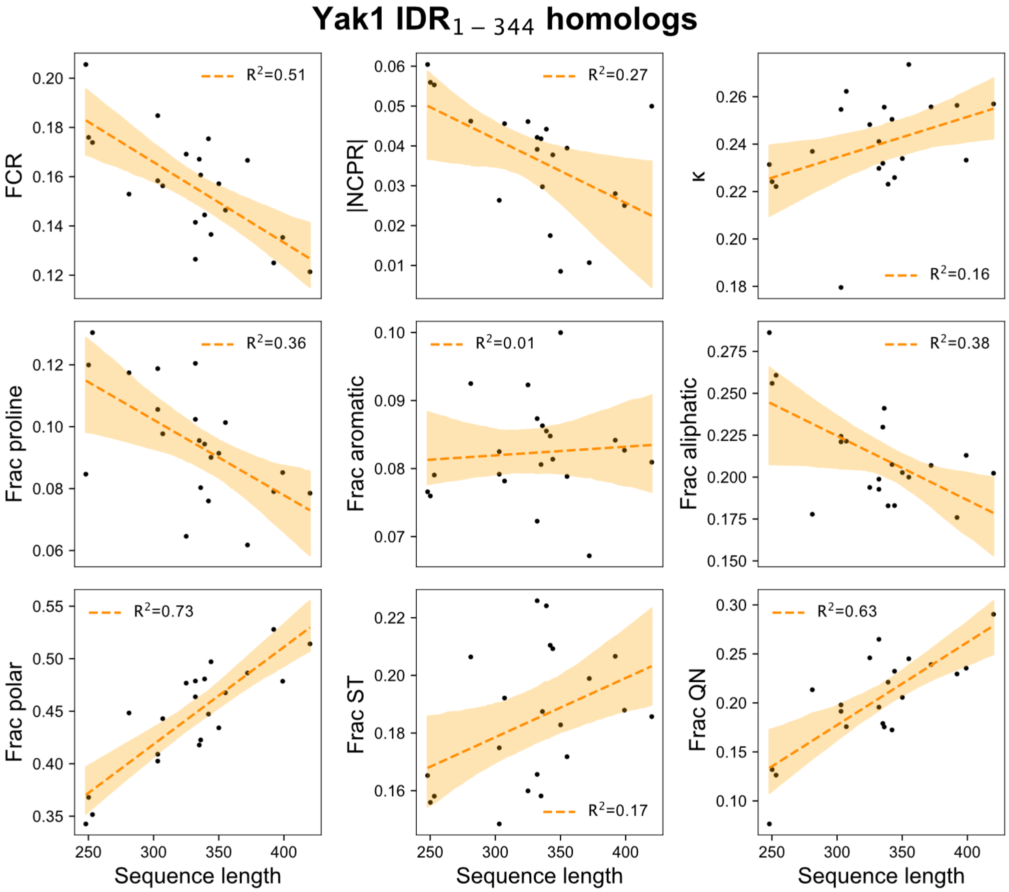

Figure S13. Expanded comparison of Yak1 IDR homologs' sequence features. Yak1 homolog sequence length compared to different protein sequence features. The line of best fit (Pearson correlation) and 95% confidence intervals determined by bootstrapping are denoted in orange. FCR is the fraction of charged residues, |NCPR|=absolute value of net charge per residue, ϰ (kappa) represents the charge asymmetry patterning parameter, Frac ST corresponds to the fraction of serine and threonine, and Frac QN corresponds to the fraction of glutamine and asparagine residues.

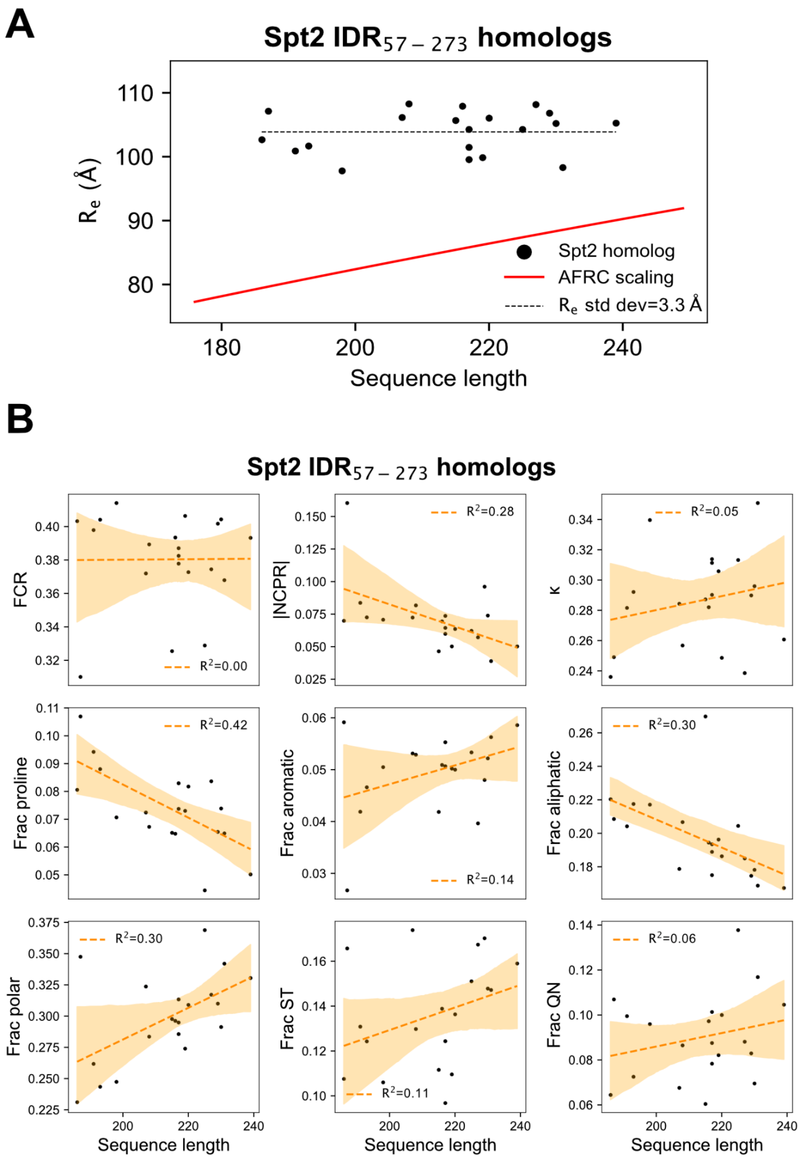

Figure S14. Spt2 IDR has conserved dimensions across homologs despite divergent sequences. A) Sequence length and ALBATROSS-predicted R_e for Spt2 and its yeast homologs. The gray dashed line denotes the mean R_e. The red line denotes R_e as a function of sequence length for an Analytical Flory Random Coil polymer scaling model, a null model shown to represent expected scaling if amino acid sequence had no major impacts on chain dimensions. **B)** Spt2 homolog sequence length compared to different protein sequence features. The line of best fit (Pearson correlation) and 95% confidence intervals determined by bootstrapping are denoted in orange. FCR is the fraction of charged residues, |NCPR|=absolute value of net charge per residue, ϰ (kappa) represents the charge asymmetry patterning parameter, Frac ST corresponds to the fraction of serine and threonine, and Frac QN corresponds to the fraction of glutamine and asparagine residues.

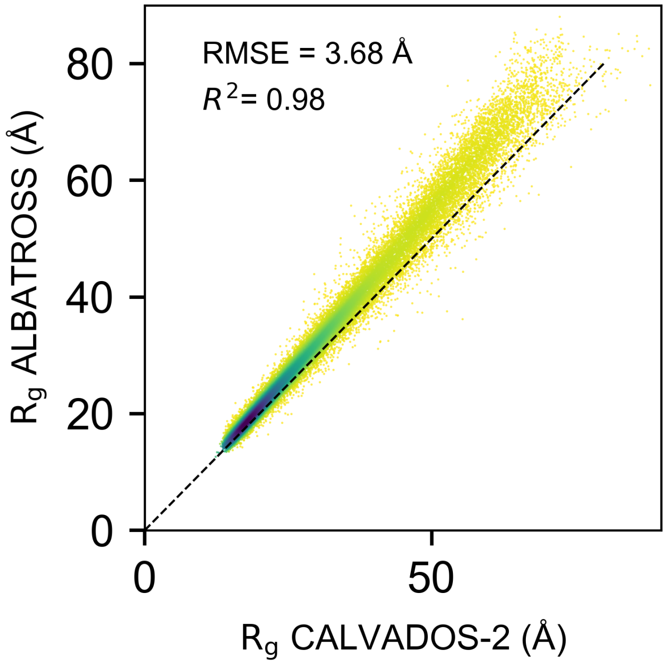

Figure S15. **Comparison of ALBATROSS predictions and R_g values obtained from Tesei & Trolle *et al.* IDR-ome**[17]. The root mean square error here is equivalent to the mean square error between Mpipi-GG and SAXS data (see **Fig. S8**). ALBATROSS R_g values are systematically slightly more expanded than CALVADOS2 values. This difference is in part explained by the proline-drive expansion seen in Mpipi-GG and hence ALBATROSS. However, this may also reflect a slight underestimation of hydrophobic interactions in Mpipi-GG and, hence the resulting ALBATROSS predictions.

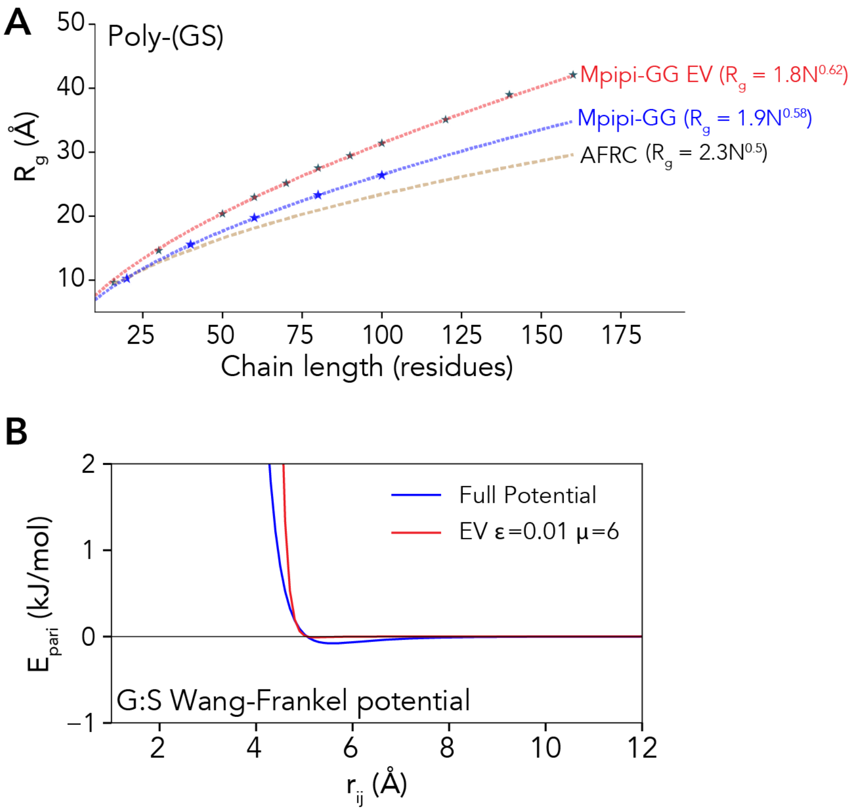

Figure S16. Parameterizing an excluded volume model for the Mpipi-GG force field. A) Scaling behavior for poly-GS as represented in terms of Mpipi-GG (blue) Mpipi-GG as an excluded volume (EV) simulation and the Analytical Flory Random Coil (AFRC) model. EV simulations are more extended than full Mipi-GG simulations. **B)** Wang-Frankel interaction potentials for a representative pair of beads (G:S). The full Wang-Frankel potentials for the interaction of glycine and serine in the Mpipi-GG forcefield - note the dip near r_{ij} of ~5.5, reflecting the attractive part of the potential. After tuning the $\boldsymbol{\sigma}$ and $\boldsymbol{\mu}$ parameters, we obtained a pairwise interaction potential with near zero attractive interactions (red) that match the same dimensions as the full Mpipi-GG forcefield.

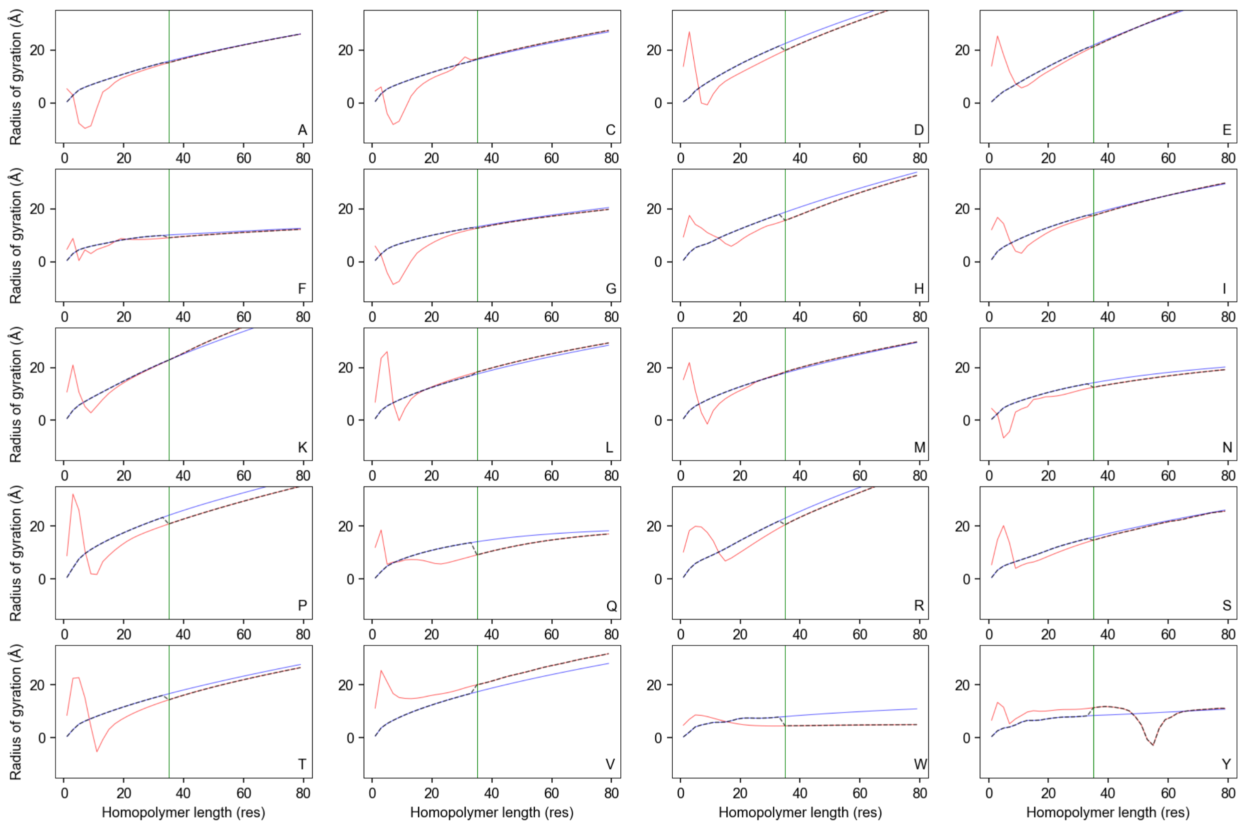

Fig. S17. Comparison of the scaled radius of gyration networks (blue) vs. non-scaled radius of gyration networks (red) for a set of 20 homo-polymeric sequences. A reasonable agreement between the two models is obtained above a length of 35 amino acids (green vertical line), with some exceptions (notably poly-tryptophan and poly-tyrosine). However, below this threshold, we often see substantial deviations between the scaled and non-scaled networks, with non-scaled networks showing unphysical behavior. Based on these observations, in ALBATROSS V2, we use a threshold of 35 residues, and, by default, even if a non-scaled network prediction is requested, we fall back to using the scaled network prediction to avoid nonsensical R_g and R_e values. Encouragingly, the agreement between the two models above 35 amino acids is much closer for non-homopolymeric sequences (see **Fig. S18**).

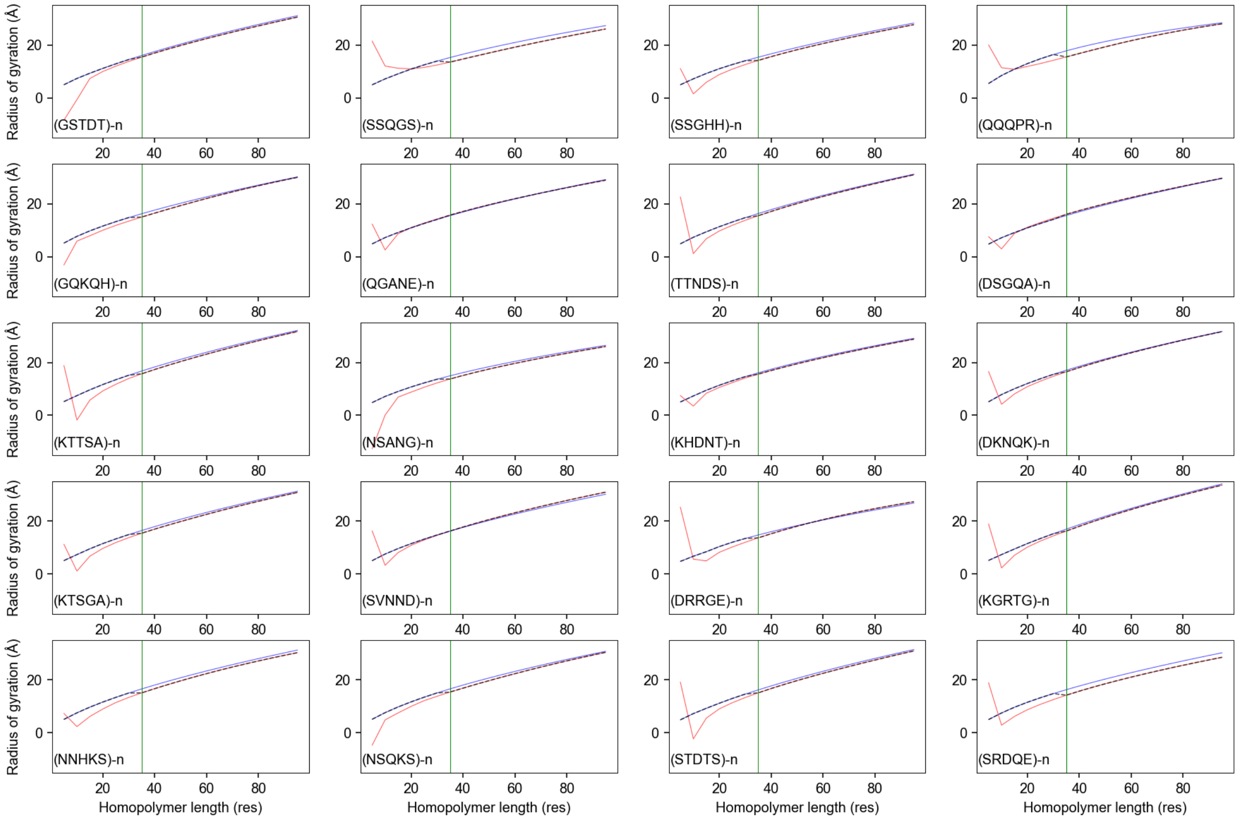

Fig. S18. Comparison of the scaled radius of gyration networks (blue) vs. non-scaled radius of gyration networks (red) for a set of 20 random disordered repeat proteins. Above a length of 35 amino acids (green vertical line), good agreement between the two models is obtained, but below this line, we often see substantial deviations, where the non-scaled network (red) shows unphysical behavior.

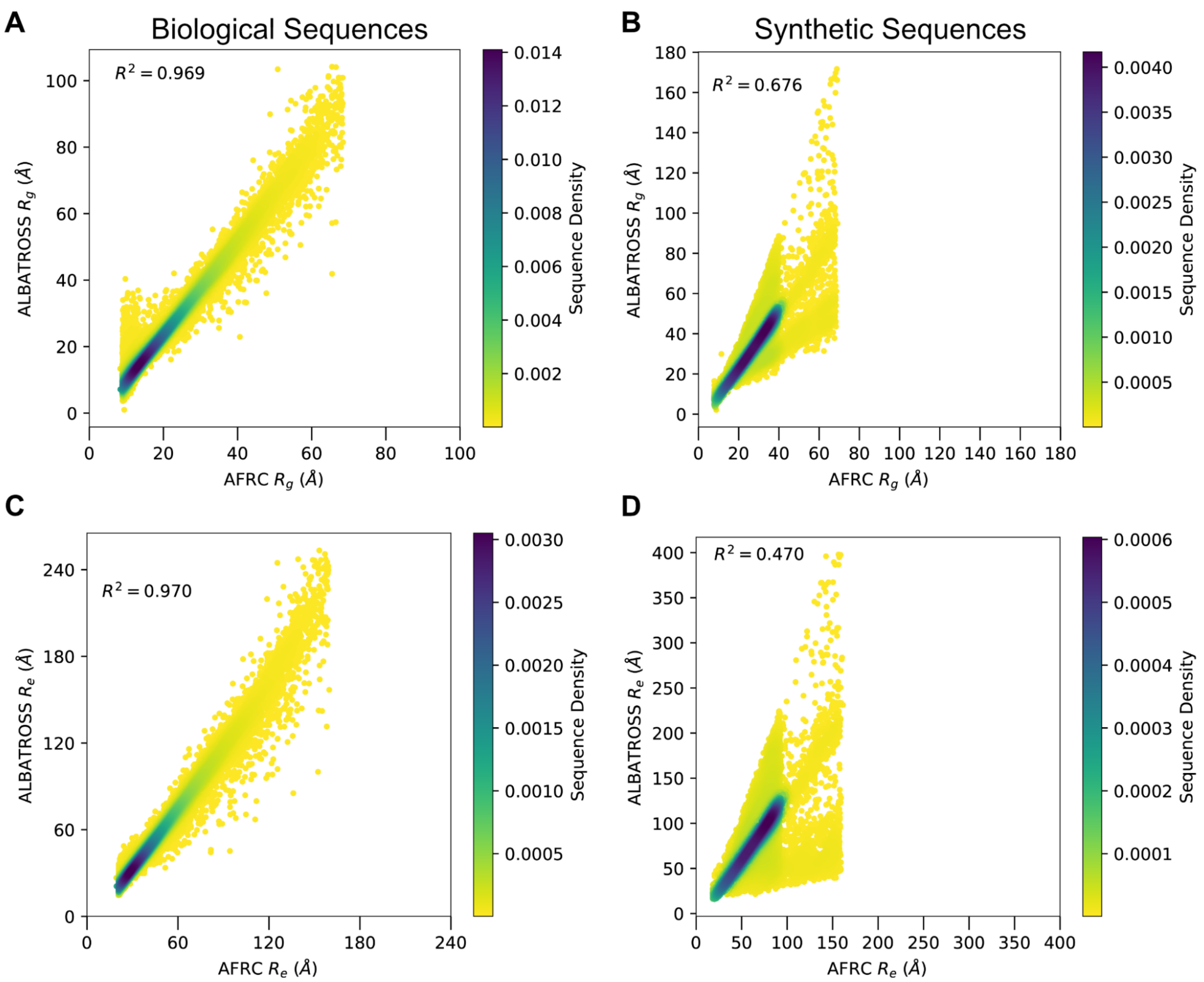

Figure S19. Comparing the Analytical Flory Random Coil (AFRC) chain dimensions and the ALBATROSS predicted chain dimensions for the synthetic and biological sequence libraries. A-B) Correlations between modeled radii of gyration for both biological sequences (right) and the synthetic sequences (left). **C-D)** Correlations between modeled end-to-end distances for both biological sequences (right) and the synthetic sequences (left).

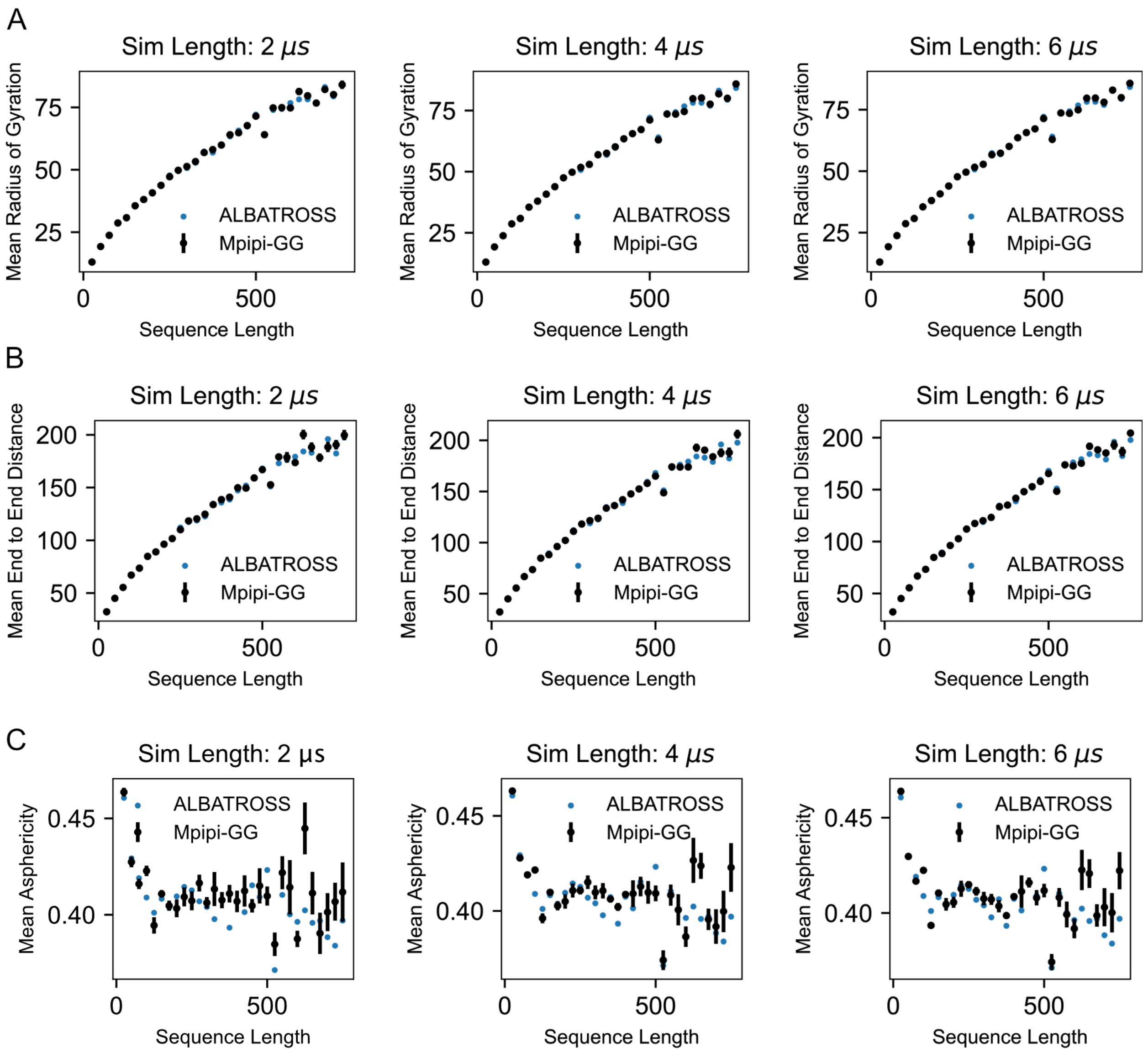

Figure S20. Comparing computed ensemble average properties as a function of simulation time. A-C) The calculated mean radii of gyration, end-to-end distance, and asphericity as a function of sequence length from different amounts of simulation data. The first column represents the mean observable from 2 microseconds of simulation data; the second column corresponds to the mean observable computed over 4 microseconds of simulation data, and the third column corresponds to the mean observable computed over 6 microseconds of simulation data. The standard error of the mean from N=5 simulation replicates is shown in all scenarios. The observed standard error bars are small in most cases. In all plots, the predicted ALBATROSS property is shown in blue for comparison, although often blue and black marks overlap perfectly.

3. Supplementary Tables

Table S1. Table showing proteins with overly-compact subregions ordered by protein abundance.
Protein abundance data is from quantitative proteomics mass spectrometry experiments previously published by Hein et al. and parsed and built into SHEPHARD-compliant protein attribute annotation files[24,25]. See separate .xlsx file, or data individual file at the GitHub repository.

Table S2. Table showing proteins with overly-expanded subregions ordered by protein abundance.
Protein abundance data is from quantitative proteomics mass spectrometry experiments previously published by Hein et al. and parsed and built into SHEPHARD-compliant protein attribute annotation files[24,25]. See separate .xlsx file, or data individual file at the GitHub repository.

Table S3. Gene ontology analysis for proteins with compact IDRs. For annotations that contained 100 or more entries in the basis set and showed 2-fold or higher enrichment, the vast majority of annotated terms pertain to RNA in some way across the three classes of gene ontology. PANTHER reports using Fisher's exact test (default behavior, two-sided). See separate .xlsx file, or data individual file at the GitHub repository.

Table S4. Gene ontology analysis for proteins with expanded IDRs. For annotations that contained 100 or more entries in the basis set and showed 2-fold or higher enrichment, a variety of IDR-associated annotations are identified, including chromatin binding, cytoskeletal regulation, and cellular organization, in good agreement with analogous analysis from Tesei & Trolle, despite the two analyses being done in different ways [17]. PANTHER reports using Fisher's exact test (default behavior, two-sided). See separate .xlsx file, or data individual file at the GitHub repository.

Organism	ID	Res. #	Sequence	Pred. R_e (Å)
S. cerevisiae	YJL141C	1-344	MNSSNNNDSSSSNSNMNNSLSPTLVTHSDASMGSGRASPDNSHMGRGIW<br>N<br>PSYVNQGSQRSPQQQHQNHHQQQQQQQQQQQQNSQFCFVNPWNEEKVTN<br>S<br>QQNLVYPPQYDDLNSNESLDAYRRRKSSLVVPPARAPAPNPFQYDSYPA<br>Y<br>TSSNTSLAGNSSGQYPSGYQQQQQQVYQQGAIHPSQFGSRFVPSLYDRQ<br>D<br>FQRRQSLAATNYSSNFSSLNSNTNQGTNSIPVMSPYRRLSAYPPSTSPP<br>L<br>QPPFKQLRRDEVQGQKLSIPQMQLCNSKNDLQPVLNATPKFRRASLNSK<br>T<br>ISPLVSVTKSLITTYSLCSPEFTYQTSKNPKRVLTKPSEGKCNN	111.9

V. polyspora	1060.53	1-303	MQGEVMSNNRPFDNGNHNGNSNNNEEKKDFWNPAFMTQQRGFQQQQQQ<br>P<br>QQSALNSDIDPGMRPGQFIFNNPWRNDPASRKNSLAASPFPDNSMGMLD<br>I<br>DYRRRKSSLVTPPSRTANTNPFQSDKAANFPQSTAAYQQQRQFLANDNG<br>M<br>FADRLGPSMNFVDPNDFYRRQSVAAVSYQPDTGPTITANQSIPITHYRR<br>L<br>SAYPQSMTPSLHPIRREEPLVKQQPIHIPQMHITNSANDLQPLINPTPD<br>Y<br>RRASLNSKEISPLNALTTGLITTYSLCSPDFAYKTSKNPKRVLTKPNEP<br>R<br>GNN	111.3
V. polyspora	2001.10	1-350	MKADSSSNTNDPNSDNENDKQNMWNPIFINQNPVRYMSQQQQHQYQHL<br>P<br>QNLQHQQQQLTQEYSNSQQMAFVNPWMAATDDSINYSPLYSTAPPSTL<br>S<br>VLQESDEHPQKIPCFDNYNYDQRRKSSIIIPPTREPAPNPYLQEYYNGY<br>P<br>APVFQLETNEMMNQQQQYNPNYFICNQPLYQQSGDSTFSIADPTMSFSN<br>Q<br>DLTMRQSVGPEHFISDHTNSNQYSKNGKSTQQLISYRRLSAFPQTNGFH<br>T<br>LQPPMYISSEYRKSSMSVSPKPLMISHLKQCHSKADLEPVMNQTPKFRR<br>A<br>SLNSKTISPLIALTKGLITTYSLCSPSFSYKTCKNPKRVLTKPSDGKFN<br>N	112.3
T. phaffii	TPHA0A0457<br>0	1-325	MEVDNEEISNGGGFWNPKYLEQNDSNGSGGNNNNNNDNNNSQGIQWNQN<br>F<br>LDSQPNVAASQVNSNFNMKRRKSSIIITPPSRTANTNPFQNKNIISDGN<br>Y<br>NNSYIRNYDTTRRGSIAGYNYSHVSNNSVRFPQQQLAPQQQQQSYYNQP<br>P<br>LGGYSLQKNLHRSKVKGFIDPNDLQRRQSVATVNYDTKQHINNINPNTG<br>K<br>PSVQNLHGSFNFRRRQSAYPIFNDSFAYNNSSGGNKKFSIQKNDPINLI<br>P<br>TMFNVQSKSDLKPTLNILKDDTRRASINSQKISPLHALTSGLVTTYSLC<br>S<br>PDFQYNLSKNPKRVLTKPSEPAYNN	103.5
T. blattae	TBLA0B06510	1-342	MENQTIQEYPENNSEEGAPNDTSYKTPLQHRGSQSYSHQQLHNGQRNSL<br>T<br>FINPWGSNLNGSSDLFMQSNQSLLSTSSENQRRPSMDDFKRRKSSIVIP<br>P<br>SRAPGINPYFYNIDTTATFNGDGTSDNFQQNQNIDANQQQKKIFADEKD<br>L<br>YVQALLDPSSGIYDPDAFYRRRSLAAPAFTSSMGSSSMNTANNTATTNA<br>S<br>SYQLNEPISPSNVNFSLMPHGCGINSRQPNRTGSFRKLSAYGAPVQSTF<br>K<br>STYREEYNLLQQPPLEIPQMTPCNSKSDLQPTLNKLPKYRRASLHSSTI<br>S<br>PLVGLTKGLITTYSLCSSDFQYKTSKNPRRVLTKPNEGVANN	120.9
T. blattae	TBLA0D05770	1-372	MQRRYSELDPEDMDINSRSNSNSSSNSLHNNAMTTLMQESSTKMQFKNQ<br>H<br>RFNNPWGNGSNNNNDTFDYLGSRKEFDSMEHHVSIMEPEELTGNNSVGS<br>I<br>TCSDINNEQYKRRKSSVIIPPTREAGPNLYQHLMNKNWQATSNNNGNNN<br>N<br>LNMNNNLFQNMQPNSTTTSNVNISLNMNTNMNFHMQNPDVNSRFLANED<br>S<br>FRRQSLATFNYNPQHTATNLHSHYNPNMVNSANPTNNNVLLRNPSTNST<br>T<br>TSPYRRMSTFPQNINPLASFNNQVLKDENQLHLLQQSQSQSQQPIIIPH<br>M	119.7

			TKCTNKSDLQPIINDQPEYRRASTFTNNVSPLKSLTTRLITTYSLCSKD<br>F<br>IYKTSRNPKRVLTKPCEPVEND	
N. dairenensis	NDAI0G06240	1-420	MESIHNGTMSSNGNAAVQITNNDNNLKNNSPFNNSIWKANYVENSQQQQ<br>Q<br>QQQQQLQSKDSLTPGGVSPARKQQGMGPPSSIPQQQQQQQQQQFSFTNP<br>W<br>NKNDTVLPQQTYNYAMDEDMLESFKRRKSSLVIPPSRARAPNPFEFNFQ<br>Y<br>QAFPQQSSQQGINNNSNSNPYSYQQYQQHQLYNTHQYNNPNLMAANGNN<br>N<br>SRFFNQQDFHRRQSVAASQFYPSTNTSNISNNTKSMMNSPNSVSMQSNN<br>N<br>NIHSSNTYLPTATPTNTSNNNIMVNNNNNNNNNNNNNNNNNYNPNSAIT<br>A<br>SFRRLSAYPTTGISSTIPSQQQLLRKGSILEASPSAAATAQPIVIPTMK<br>K<br>VNFKKDLKPIINPTPKFRRASLNSKTISPLLALTRNLITTYQLCSPDFT<br>Y<br>KPSKNPKRVLTKPSEGKFNN	121.2
N. castellii	NCAS0I02680	1-355	MTTPNDFASPKEMSPRHANSSESLHNNHQLQQGAADQANNNNNIWKSTF<br>M<br>NQQQQQQQQQNSFAFTNPWSSNNADSTLSPPQQQQQSYNMDMDEDMLES<br>F<br>KRRKSSLVIPPSRAPAPNPFQYDQYNQYGPPQSQQQVPSQQQEINYSHQ<br>Q<br>QQQLYNAHQYGARLGPSMYQDFQRRQSMAASHLYPNGNGNNITNYSTRT<br>I<br>NKNSSITPNTANPSMMAGPSYIPGGLSNDNSMVSPFRRLSAYPTTNIHT<br>P<br>ATANLQPPYKQLRRTGEEQQQQQQPKPLIIPSMKVCKNRNDLNPITNPT<br>P<br>KFRRASLNSKTISPLLALTKNLITTYQLCSPDFTYKPSKNPKRVLTKPS<br>E<br>GKFNN	118.2
K. naganishii	KNAG0C0260<br>0	1-392	MHQNSSQMTNNNGNSIWNPNFFQNDEGQQQQQEQQQQQQQQQQKQEVKF<br>A<br>FNNPWDSNGGNQNDYSTGQVAGDAAGNTGHVLYSPNHEMIMETPNEDFE<br>N<br>FKRRKSSLVIPPTRAGAPNPFQYENYPTYNSMGQNTSSINSNGDYGPGG<br>A<br>SSSAGPGTALSGFNPSMGSSQQQRQSLFNKQQQQQQHLNTRFVPNLYNQ<br>Q<br>NGFQRRQSMAATAFTTPQYTTAPSTAGNTTSNFGNNAGTVGPNGHFSTN<br>M<br>ANSSSAGKLQNVQSGMNPNFMGMSSVSPYRRLSAFPTSSGGMTSATPTT<br>S<br>TPFPQQFAPLREEKPTIPQMSSCKSKSELSPTVNKTPKYRRASVHSQTI<br>S<br>PLNAMTKNLITTYQLCSPDFIYKTSKNPKRVLTKPSEGKCNN	119.1
K. africana	KAFR0F0194<br>0	1-399	MDQTNGQQMNSPFDNSGNTNNSTNNTNNNTNNNGNNDGSDNGYHLWNQD<br>Y<br>LSPGHPQVSLDSQMNLQNNHIQQQKNEKQHTPTFIFTNPWDNNEKIPQQ<br>N<br>LQYSQNYETYGMSNEDFEAFKRRKSSLVIPPARAPAPNPFHYDKYPLYS<br>N<br>INNNNINNSHRPSLGSSSSSLAPMNLQMSNPSLYQQQAQQAQDLFEKQQ<br>A<br>QQQLLSSRFLPSFYNAHNQDAQRRQSLAAPYYSQTYQSNPSVAGSKANS<br>I<br>TPSNAIPGSNSTNNYSTNAPNMTSLPAILPYRRLSAYPSSTAHLLHPSN<br>L<br>RMAATDADLLQQHLYPRPQSQYTVPSLQKCSSKSDLQPIVNPTPKFRRA<br>S<br>LHSKTISPLVGLTKNLITTYQLCSSDFTYKTSKNPKRVLTKPNEAKLNN	119.9

C. glabrata	CAGL0I05896g	1-336	MVQIYYNENQALEPMQHNQANGIWKASYMDEANADSKRFAFTNPWGNEK<br>I<br>DEDEAMSYNYSSANSTADVDMNSDMYKRRKSSLVVPPSRAAAPNPFQYE<br>T<br>MQNSTGTAQRAMYPESSAYQQQRQIMVPHQQQGQLASRFNSRFVPSLYN<br>P<br>QEFQRRQSLATAQFSTPSSLHGSASNQAFNTGSGNNGVNLNYASANVTT<br>N<br>NSSTYLSAMTPNVPMEMGNIPLASPHRRLSAFPTSGTTNSSLQPPFKQL<br>R<br>RDEGSPRQIVIPQMHRCHSKNDLQPCINPNPKFRRASLHSNTISPLIGL<br>T<br>KSLLTTYALCSPDFTYQTSKNPKRVLTKPSEGKYNN	116.7
S. uvarum	6.221	1-281	MSQTSQRPPQQQQQHSQQQQNPQFSFVNPWGEEKITNSQQNLVYPPQYD<br>D<br>PNTNESLDAYRRRKSSLVVPPTRAPAPNPFEYDSYPAYTNSNTSLPANG<br>Q<br>FSSAYQQQQQQQVYQQSTIHPSQFGSRFVPSLYDRQEFQRRQSLATTNY<br>S<br>SNFPSINSNANQGTSSIPAMSPYRRLSAYPPSTSPPLQPPFKQLRRDEI<br>Q<br>GQKLSIPQMQPCNSKNDLQPVINATPKFRRASLNSKTISPLVSVTKSLI<br>T<br>TYSLCSPDFTYQTSKNPKRVLTKPSEGKCNN	105.8
S. kudriavzevii	10.73	1-332	MNTSNNNDSTSSNGKNTSLSPTLATHSDASMGSGGASQDTSHLGSSIWN<br>P<br>SYMNQSSQRPLQKQQQLQQQQNPQFCFVNPWNEEKVTNSQQNLVYPPQY<br>D<br>DLNTSESLDAYRRRKSSLVVPPTRAPAPNPFQYDSYPAYTSSNTSLPAN<br>N<br>GGQYPFAYQQQQHAYQQGAIPPSQYGTRFVPSLYDRQEFQRRQSLAATN<br>Y<br>SSNFSSVNSNANQGTSSIPTISPYRRLSAYPPSTSPPLQPPFKQLRRDE<br>V<br>QAQKLSIPQMQPCNSKSDLQPVLNATPKFRRASLNSKTISPLVSVTKSL<br>I<br>TTYSLCSPDFTYQTSKNPKRVLTKPSEGKCNN	117.6
S. mikatae	10.91	1-339	MNTSNNNDSTSTNSNKNASLSPTVATNSDASVGSGRASQDNSHLGSSIW<br>N<br>PSYVNQSSQRHPQQQQQQQQNSQFCFVNPWNEEKVTNSQQNLVYPLQY<br>D<br>DLNSNESLDAYRRRKSSLVVPPARAPAPNPFQYDSYPAYTSSNTNLPGN<br>S<br>SGQYPSAYQQQQQQRQQQHAYQQGTIPPSQFGSRFVPSLYDRQEFQRR<br>Q<br>SLAATNYSSNFSTFNSNANQGTSSIPVISPYRRLSAYPPSTSPPLQPPF<br>K<br>QLRRDEVQAQKLSIPQMQPCSSKNDLQPVSNATPKFRRASLNSKTISPL<br>I<br>SVTKSLITTYSLCSPDFTYQTSKNPKRVLTKPSEGKCNK	115.2
Z. rouxii	ZYRO0D02970g	1-335	MWNPLYLSSEDQEQLKKQQQQGLQQRLHQMERRQSQSQRAQHQQHSQQ<br>H<br>QQPQQQVQFTFTNPWGNNEDLDPYYSSSAGASSTGLDTSETNPLLAAEY<br>G<br>GDGFDTYRRKSSLVIPPARAPNPNPFEYDDRNAYAGVYDPQRQNQIRNM<br>L<br>LAQQQQQLAGARFVPSSFYGAGGDLHRRQSVAAVHYPPSGPTNLASLGA<br>P<br>TNFTPAAGAATNMTPYRRLSCYPPSTSPTASLQPPYKQLRRDAGAQGAM<br>V<br>GAPSPQQVKIPQMQKCNSKSELKPTLNATPKYRRASLNSKTISPVIALT<br>K<br>GLITTYSLCSPDFSYQTSKNPKRVLTKPNEGKYNG	117.3

T. delbrueckii	TDEL0H0089<br>0	1-303	MQQGAEATGNEEANNGNQSIWNPLFMSQEENVQQLPPPQGRQQVQFNFV<br>N<br>PWGVANGENAQVNQDNVPDETAYLTPSDKENVDSFARRKSSLVIPPARA<br>P<br>GPNPFEYDDAKAYPNMYSQRQRPMFFPQQQQQLPQQTYNSGRFVPSGFY<br>N<br>PQDSQRRQSVAVVHYPTTAPNTSNASTMIPATGATHMNSPNRRLSAYPP<br>S<br>TSPTTSLQPPYKQLRRDQGTAPPQQIIIPQMQRCTSKTDLSPMVNATPK<br>F<br>RRASLNSTTMSPLIALTKNLITTYSLCSPDFSYQTSKNPKRVLTKPSEA<br>K<br>CNS	117.0
K. lactis	KLLA0A05819<br>g	1-307	MGDKNSLWNPAFMASQQGASSSNQNQNFSQTQANTQNQGPEIAPDGPAG<br>S<br>SSNAGGNIYAGQQQGGNAAGGFQMQSPRQSIFKNPWQEIPQIPESQRST<br>M<br>RKQSNFPLPTTYEEDNAVDESSQQFRRRNSSLVIPPARAAGPDPFLYEK<br>Q<br>PFPNYNDAQRRQSVAVTGGSQLSPQQVTKPSQLRKPGINTAGYMAGSIN<br>S<br>SVYPATAGAYMSTRGGSSSLPSSRPPSPIIIVPKFNKIFTRQDLNPVIH<br>S<br>TPKYRRASLSSKTVSPLMALTKSLTTTYSLCNDDFAYQTSKNPKRVLTK<br>P<br>NEGKYNN	121.0
E. gossypii	ACR249C	1-248	MKEERKSIWNPAFDMSAGAAKTGKVLGRQSFRNPWGEPGSPAGRRQSGF<br>Q<br>QLATTFEEGQGEEERARRRSSLIVPPTRAAGPEPLLRETYGDSWGYGSE<br>F<br>QRRQSVAVAGTYHSPGYFETGAQQPSTSPSLMVAHAVPYRKLSAYPPLA<br>G<br>ALVPAASLNSTLRGSAGGVAAAVPQMRKVGARQELAPVLHAMPKFRRAS<br>L<br>NSKTVSPLIALTKSLIITYSLCSDEFSYQTSKNPKRLLTKPSEGKLNN	110.6
L. kluyveri	SAKL0C0624<br>8g	1-332	MNNPVGKAQEDGTTQFQSQLPPQPLQEPQQQPQQQPQQQPQQQPQQQPQ<br>Q<br>QPQQQPQQQSIWNPAFMSNASGSTTPQGQQTFPNPWTDSTNSIHASPAQ<br>R<br>RLSGFNQQLPTTYEAVQADSQSNFRRRNSSLVIPPTRAAGPDPFLYDAQ<br>Q<br>QQQQQIFPFYHQLQQYSINQDAQRRQSVAVAGSYNRQSMGYLTEGTNYM<br>I<br>QQPQQLLQQQQQQQQPQQPQQGHSPYRRLSAYPVTAGTVLPPPFKQIRR<br>D<br>SSTPVAIPRLHRVSARQDLRPVINATPKHRRASLNSKTVSPLVALTTSL<br>T<br>TTYTLCSPDFSYQTSKNPKRVLTKPSEPKYNN	110.0
L. thermotolerans	KLTH0F05522<br>g	1-250	MPESDKSIWNPAFLNNAQKQQGAPSYGFKNPWHDVSGATGKRNSMQFNQ<br>H<br>LPTTYEEAPQHGSASENGFRRRNSSLIVPPPRAAGPAPDAYMYGMQFVP<br>Q<br>QGYPGNSQELHRRQSVAVAPQHSFAQPALPADMSMHAPMSPFHRKLSSY<br>P<br>ATAGSVLPPPVKQMRRQDEMIPVVLPQMHKVNARQDMRPTINATPKYRR<br>A<br>SLDSRTVSPLVALTKSLTTTYTLCSPEFSYQTSKNPKRVLTKPSEGKNN<br>N	109.3
L. waltii	33.13984	1-253	MPDSDKSIWNPAFLSNSQKQPASPAYGFKNPWNDATSAAAKRNSMQFSQ<br>Q<br>LPTTYEDIQHNAGMSENEFRRRNSSLVVPPPRAAGPGPDAYMYGMQFVP<br>P<br>QGYPPYAQELHRRQSVAVAPQHTQHAFTQPSSAVDHPMHAPMSPFHRKL	110.1

			S<br>SYPPVAGSVLPPPVKQLRRQEEVMPVVLPQMHKVNTRQDLRPAINATPK<br>Y<br>RRASLNSKTVSPLVALTKSLTTTYALCSPDFSYQTSKNPKRVLTKPSEG<br>K<br>HNN	

Table S5. Yak1 homologs. Yak1 homolog IDR sequences, along with organism, reference ID, residue positions, and ALBATROSS-predicted R_e.

Organism	ID	Res. #	Sequence	Pred. R_e (Å)
S. cerevisiae	YER161C	57-273	QELLKNGALAKKSGVKRKRGTSSGSEKKKIERNDDDEGGLGIRFKRSIG<br>A<br>SHAPLKPVVRKKPEPIKKMSFEELMKQAENNEKQPPKVKSSEPVTKERP<br>H<br>FNKPGFKSSKRPQKKASPGATLRGVSSGGNSIKSSDSPKPVKLNLPTNG<br>F<br>AQPNRRLKEKLESRKQKSRYQDDYDEEDNDMDDFIEDDEDEGYHSKSKH<br>S<br>NGPGYDRDEIWAMFNRG	99.6
V. polyspora	1025.5	58-288	QELIKSGKLNPKGTSNKSRSSSSTPGGKRKNSPSDNDNGTEFKFKRKIN<br>S<br>NNLPKFQKPVETKHAPLKKMSFDELMKQAENNAHSKPESEPVLNSKSKE<br>L<br>NVGKSVQQKYRISKQGFKSNRNERMHNSTSVRNSRPSPKPHTTSHDLKP<br>V<br>IVPIPKGGGLAKPNEKLRERLEMKKQKLRRGRYEDEEEDEDDYDDDMDD<br>F<br>IDDDEEDYSSVSRSHKANYNRDEIWAMFNKG	98.3
T. phaffii	TPHA0B03460	58-282	KENLKNNITKNKAASRSATLTRQRSSTTSKIDNSTETTFKRKPGQNTKA<br>F<br>SNQGQLKKKPTALKKISFEDLMKQAESNNVPNSNGDQSLKNVNNKRPLL<br>T<br>KPGFKSRKVTKPNMKNVRRSEVSVTHRAENISKKDNGPVMVKLPTMGIA<br>K<br>PNAKLIQKMKHKNSKGKGFGDRYGKSSQDHYDSEEDSDLDDFIDDDEDN<br>D<br>GYGDLEAAGDPGYNRDDIWAIFNKG	104.3
T. blattae	TBLA0F03750	55-293	KELLKNGGDVHKKSAAKKESSLKSSTTKSSSRRPKNRDSHSETTYKRKI<br>G<br>ERTKGASYQGSNNVISRKEHTKKMSFDELMKQAETNKTKPEIDMNKQNL<br>P<br>KPARRLSKPGFKPSKYARNNQIAKANSTDLNSERKSHNSNNLRDTPSLS<br>K<br>PSGEESPVVVQIPKNNFARPNEKIRKMLDSRKRSHKRQYDYDDEEEDDM<br>S<br>DFIEDDEGEEDSYHNYDRRKADRDPGYDRDEIWAMFNKG	105.3
N. dairenensis	NDAI0C06050	60-288	QERLKNGELEKKKPKPRRSPGSSSGSKSTRKRDNDDVEGSLGTVYKKKV<br>G<br>SSNAKLVPVRSLTKKLEPIKKLSFEELMKQAESNSKGGSPDNTAVKVTA<br>S<br>IKKTRPPPRISKPGFKSFKERKTTASKSTSTVNNKSKPEFSRNHPNGRH<br>L<br>KEQSAVKLKIPKNIVAQPNRMIKQKLESKRRTLESKYSNRRGRYEDQYD<br>D<br>DMDDFIEDDEEEEEENYRSKSRRDERPWL	106.8

N. castellii	NCAS0A03730	63-292	KEQIKNGEFAKKHKKTNPSTTTKRKSKKDDDNLAADGYSRFKKKLGSTH<br>T<br>RPTPVRTLTRKMEPIKKISFDELMKQAENNASSKESSEGISKKESPSAS<br>R<br>PHLHKPGFRSARDRNRVSKPVKHQTTLPRKKMSLSPIRNRPGSRDATPI<br>K<br>ISLPVAQPNQRLKQRLESKRQRPSGRDRYGRPEYDYDDEDDMDDFIEDD<br>E<br>EDSEVHRRMKLHRDDPGYDRDEIWAMFNKG	105.2
K. naganishii	KNAG0G01760	61-247	KERLKSGPTGTAVKRKRRAPGAPDEPRKRRNGADSEGGLLGTVYKRRPG<br>S<br>RQQQAATHAGAATKRDAVKKMTFEELMMQAETNAVEKPATTAPPQRTAA<br>P<br>VRNPGFKPRRRRTGPASTTKTPAEPSKPTQRRPATPRFPAQPNDLLRRR<br>L<br>KQREQREQRQQQQQQHRGATAATTPRRTLSWTTSSRT	107.1
K. africana	KAFR0B02460	58-265	KEQAINNVISKKPRARKRPSSGPKNKVAKEDGGDIGTVYKKKIGSNTTV<br>S<br>RPQVKKPAPLKKMSFEELMKQAENNATISPTVKSEAKHETSSANRIMKP<br>N<br>FKHSSSKLNPRHKIDARAKIEPGKEKPVRLSLPKNKFAQPNDRIRKELE<br>S<br>RKKHKQGYRRNELDEEDSDLSDFIEHSDDDDRYKRRTLTSYDDPGYDRD<br>E<br>IWAMFNRG	108.3
G. glabrata	CAGL0L11704g	55-270	QELLKNPELAKKKQKQVRKTPSSSKASTGKKDKNGDDNMLVSRFKRKVG<br>S<br>DKPAVPIQVKKKPQPIKKLSFEELMKQAENNQTIPVSKDTQSNGAGEKI<br>K<br>GSAKLNKPGFKTSRPKSLSPTTHINKTDHGKDKSTAKEKSEPVVKIGIP<br>K<br>FAQPNERLKKKLEMRQRVNKSRRYEDEEDDMDDFIEDDEEEYSSYRTTS<br>K<br>DPGYDRDEIWAMFNKG	107.9
S. uvarum	5.295	57-275	QELLKNAALAKKNGVKRKRGTSSGSEKKRKERNDEDEGGLGIRFKRSIG<br>A<br>SHAPLTPAVRKKPEPVKKMSFEELMKQAESNEKQPIKTKSPEPVSMERP<br>R<br>LNKPGFKSSKKPLKKASPGLASRETPSRGDNMKLAEQHKPVKLNLPTNG<br>F<br>AQPNRRLKEKLDSRKQRSRYQEDYYEEDNDMDDFIEDDEDEEEAHRSRS<br>K<br>HTDGPGYDRDEIWAMFNRG	99.9
S. kudriavzevii	5.300	57-273	QELLKNGALAKKSGVKRKRSTSSGSEKKRSERNDEDEGGLGIRFKRSIG<br>A<br>SHAPLKPVVRKKPAPIKKISFEELMKQAENNEKQPAKVKSPEPIAKVRP<br>H<br>LSKPGFKSSKRLQKKPSPGTTLHGTPSRDNGVKSPESPRPVRLNLPTNG<br>F<br>AQPNKALKEKLESRKQKSRYQDGYYEEDNDMDDFIEDDEDESYRRRSKH<br>G<br>SEPGYDRDEIWAMFNRG	104.3
S. mikatae	5.333	57-273	QELLKNGALAKKSGVKRKRNNLSGSEKIKAERNDDDEGGLGIRFKRSIG<br>A<br>SHAPLKPVMRKKPEPIKKMSFEELMKQAENNEKQPSKVMSPEPVVKERP<br>H<br>FNKPGFKSSRRPQKKLSPSTPLRGTPSKDKGMKLSESPKPVRLNLPTNG<br>L<br>AQPNRRLKEKLDSKRQKSRYQDNYYEEDNDMDDFIEDDEDEGDHRRSKH<br>N<br>NGPGYDRDEIWAMFNRG	101.5

Z. rouxii	ZYRO0B11550g	58-284	QELLKKGELSKKATASRRTSASGKGTKKDSDDDNKTVTRFKKKSSSGSS<br>T<br>GSNRPTHISVAQKKKPEPVKKMSFDELMKQAENNAHSNGSSSSKSGSTT<br>P<br>VRAPSETQPRPRPHINKPGFKNPDRRRRAGSQEPVKPVVKNQPTPTVKE<br>P<br>RPAKLSLPKKDFAKPNELIRRRLEAKKNASRVQETQQDDYESDMDDFIE<br>D<br>DEEEDRVAMEKDPGYDRDEIWALFNRG	108.2
T. delbrueckii	TDEL0A01380	58-277	QELLKSGELSKKVRGQSKPAPTRRRKDDDEGGSMGTKFKRKVRPHSAGS<br>S<br>LPTTNAKKAEPLKKLSFDELMKQAENNAQEKPNAMKEPSPVNSGPHRIP<br>K<br>SQGTTQQYVKKIGFKKGADRRNRNSTSPVPEIPTTKPYRDEPAPIKLKT<br>M<br>VNGFAKPNEKLRRQLEQRKRTIKPRTEYEDDGSDLDDFIEDDTMESENR<br>R<br>QSQRDTGYDRDEIWAMFNRG	106.0
K. lactis	KLLA0F14487g	58-244	LKQGISSTSTKPAAKARHKASSPKDEAPIYKKKPGVNTTSGKYPVVVPK<br>R<br>EPIKKLSFDELMKRAEQKSREGPKEDKKPLPKKLGFDKAAKPKAATKDA<br>K<br>PVKEPKSKVMVKSFNRFAQPNEKLAKKLKLKEKKQIMARHGDQHYDSEN<br>D<br>EDLSDFIEDDDLDECEQGSKSQPYDRDEIWSIFNKG	102.7
E. gossypii	AGR161C	62-277	QEQLRKGTLKKASSQRRSKANGGEVASGGVRQEGSTRWKLPRPKSTVVA<br>A<br>AAPAPPLKKLSFEELMKQAEEKAKSPAASGKRTAPAGPSAPAVSKPGFK<br>P<br>RSNSGAAVVGGKVAGADKGARNGGADRTAHAPNSARGMKAKQAIAIDLP<br>S<br>GGGLAKPNEKLRRILEKQERRKRSAGEYEEDDSDLDDFIADDDGEEEGG<br>S<br>YGYDKEEIWSIFNKG	105.7
E. cymbalariae	7222	62-268	QDQIKRGSLGTPRSGSRKRLKTPGASRSNKGELSEEDDKVSFTTKWKLP<br>N<br>PSKVSKPSVPKGPLKKLSFDDLMKQAEEKAKSSKPEPDVATQSSRTTTT<br>K<br>LTKRGFKDKTRHTPKALVKGFTGGSPVKKKPIEKPVKIRVPSSNGIAKP<br>N<br>EKLRKMLEKRNIKRQTTEFEGNGSDLEDFIDDEEDEVDSQDGYGYNKDE<br>I<br>WSIFNKG	106.2
L. kluyveri	SAKL0H18084g	66-263	KEQLKKGTLQTKKPSVSRKRLDDTVTEARFKRKVRSSTVTRQTLPVNRQ<br>P<br>IKKLSFDELMKQAEQKSKNPPLSPSPLSKNEKKKDVKPVGLSSKIRKNG<br>F<br>KLPHQKRPVTNAKKNVPVSKKEVPVKIALPKNNIAQPSEKLRKRLEMKQ<br>Q<br>HRKRYYGDEDEDEDDMDDFIDDDDDEEAEYVKDHGYDRDEIWAMFNKG	97.8
L. thermotolerans	KLTH0G14586g	66-258	KEQLKNAPKNKPGAPSSRKKKDENSATATETKFRRKVGQSTKPQRPVAP<br>V<br>RREPLKKLSFDELMKEAEKKASDPSTDSKAPSAQKATSSIAKPIKLNKP<br>G<br>FNKGARRTPAAAPVSKPRERKEPTVKLKQLSIPKSSIAQPGEKLRKKLD<br>S<br>IKKKRQGEAYGYEDEEDLDDFIEDDEEEEGFNRDEIWAIFNKG	101.7
L. waltii	56.23413	67-257	KEQLKNAPKNKPAAPSRKRKDENSANTETKFRRKVGESLQSRKPVAPVK<br>R<br>TPLKKLSFDELMKEAEEKSKNPSTDPIDSTSRSKALQNNPPVRLQRPGF<br>K	100.9

			SAARRDRKPLSTPKITKQKSPVESLQRLPAPRPSIAQPGAKLKRKLENL<br>K<br>KHRQTDRYRSSEEEDMDDFIEDDEEEQGFNRDEIWAMFNKG	

Table S6. Spt2 homologs. Spt2 homolog IDR sequences, organism, reference ID, residue positions, and ALBATROSS-predicted R_e.

Predictor	Learning Rate	Batch Size	Number of Layers	Hidden Size
Asphericity	0.001	8	2	45
Prefactor	0.001	4	2	15
End-to-End Distance	0.001	32	1	45
Radius of Gyration	0.001	32	1	55
Scaling Exponent	0.001	8	4	20
Scaled End-to-End Distance	0.001	4	1	45
Scaled Radius of Gyration	0.001	4	1	75

Table S7. Final Network Hyperparameters. The best-performing hyperparameters used in the final ALBATROSS implementation for each polymeric property predictor are shown below.

Table S8. Experimental and prediction data used in Supplementary Figure S8. See separate .xlsx file, or data individual file at the GitHub repository.

4. Supplementary References

1. Emenecker, R. J., Guadalupe, K., Shamoon, N. M., Sukenik, S. & Holehouse, A. S. Sequence-ensemble-function relationships for disordered proteins in live cells. *bioRxiv* 2023.10.29.564547 (2023). doi:10.1101/2023.10.29.564547
2. Wang, X., Ramírez-Hinestrosa, S., Dobnikar, J. & Frenkel, D. The Lennard-Jones potential:

when (not) to use it. *Phys. Chem. Chem. Phys.* **22,** 10624–10633 (2020).

3. Joseph, J. A., Reinhardt, A., Aguirre, A., Chew, P. Y., Russell, K. O., Espinosa, J. R., Garaizar, A. & Collepardo-Guevara, R. Physics-driven coarse-grained model for biomolecular phase separation with near-quantitative accuracy. *Nat Comput Sci* **1,** 732–743 (2021).
4. Martin, E. W., Holehouse, A. S., Grace, C. R., Hughes, A., Pappu, R. V. & Mittag, T. Sequence Determinants of the Conformational Properties of an Intrinsically Disordered Protein Prior to and upon Multisite Phosphorylation. *J. Am. Chem. Soc.* **138,** 15323–15335 (2016).
5. Auton, M., Holthauzen, L. M. F. & Bolen, D. W. Anatomy of energetic changes accompanying urea-induced protein denaturation. *Proc. Natl. Acad. Sci. U. S. A.* **104,** 15317–15322 (2007).
6. Gibbs, E. B., Lu, F., Portz, B., Fisher, M. J., Medellin, B. P., Laremore, T. N., Zhang, Y. J., Gilmour, D. S. & Showalter, S. A. Phosphorylation induces sequence-specific conformational switches in the RNA polymerase II C-terminal domain. *Nat. Commun.* **8,** 15233 (2017).
7. Yarawsky, A. E., English, L. R., Whitten, S. T. & Herr, A. B. The Proline/Glycine-Rich Region of the Biofilm Adhesion Protein Aap Forms an Extended Stalk that Resists Compaction. *J. Mol. Biol.* **429,** 261–279 (2017).
8. Crick, S. L., Jayaraman, M., Frieden, C., Wetzel, R. & Pappu, R. V. Fluorescence correlation spectroscopy shows that monomeric polyglutamine molecules form collapsed structures in aqueous solutions. *Proc. Natl. Acad. Sci. U. S. A.* **103,** 16764–16769 (2006).
9. Sørensen, C. S. & Kjaergaard, M. Effective concentrations enforced by intrinsically disordered linkers are governed by polymer physics. *Proc. Natl. Acad. Sci. U. S. A.* **116,** 23124–23131 (2019).
10. Moses, D., Guadalupe, K., Yu, F., Flores, E., Perez, A., McAnelly, R., Shamoon, N. M.,

Cuevas-Zepeda, E., Merg, A. D., Martin, E. W., Holehouse, A. S. & Sukenik, S. Structural biases in disordered proteins are prevalent in the cell. *bioRxiv* 2021.11.24.469609 (2022). doi:10.1101/2021.11.24.469609

11. Moses, D., Yu, F., Ginell, G. M., Shamoon, N. M., Koenig, P. S., Holehouse, A. S. & Sukenik, S. Revealing the Hidden Sensitivity of Intrinsically Disordered Proteins to their Chemical Environment. *J. Phys. Chem. Lett.* **11,** 10131–10136 (2020).
12. Holehouse, A. S., Garai, K., Lyle, N., Vitalis, A. & Pappu, R. V. Quantitative assessments of the distinct contributions of polypeptide backbone amides versus side chain groups to chain expansion via chemical denaturation. *J. Am. Chem. Soc.* **137,** 2984–2995 (2015).
13. Holehouse, A. S. & Pappu, R. V. Collapse Transitions of Proteins and the Interplay Among Backbone, Sidechain, and Solvent Interactions. *Annu. Rev. Biophys.* **47,** 19–39 (2018).
14. Alston, J. J., Ginell, G. M., Soranno, A. & Holehouse, A. S. The Analytical Flory Random Coil Is a Simple-to-Use Reference Model for Unfolded and Disordered Proteins. *J. Phys. Chem. B* **127,** 4746–4760 (2023).
15. Vitalis, A. & Pappu, R. V. in *Annual Reports in Computational Chemistry* (ed. Wheeler, R. A.) **5,** 49–76 (Elsevier, 2009).
16. Lalmansingh, J. M., Keeley, A. T., Ruff, K. M., Pappu, R. V. & Holehouse, A. S. SOURSOP: A Python Package for the Analysis of Simulations of Intrinsically Disordered Proteins. *J. Chem. Theory Comput.* **19,** 5609–5620 (2023).
17. Tesei, G., Trolle, A. I., Jonsson, N., Betz, J., Pesce, F., Johansson, K. E. & Lindorff-Larsen, K. Conformational ensembles of the human intrinsically disordered proteome: Bridging chain compaction with function and sequence conservation. *bioRxiv* 2023.05.08.539815 (2023). doi:10.1101/2023.05.08.539815
18. Portz, B., Lu, F., Gibbs, E. B., Mayfield, J. E., Rachel Mehaffey, M., Zhang, Y. J., Brodbelt, J. S., Showalter, S. A. & Gilmour, D. S. Structural heterogeneity in the intrinsically disordered RNA polymerase II C-terminal domain. *Nat. Commun.* **8,** 15231 (2017).

19. Boze, H., Marlin, T., Durand, D., Pérez, J., Vernhet, A., Canon, F., Sarni-Manchado, P., Cheynier, V. & Cabane, B. Proline-rich salivary proteins have extended conformations. *Biophys. J.* **99,** 656–665 (2010).

20. Bremer, A., Farag, M., Borcherds, W. M., Peran, I., Martin, E. W., Pappu, R. V. & Mittag, T. Deciphering how naturally occurring sequence features impact the phase behaviours of disordered prion-like domains. *Nat. Chem.* **14,** 196–207 (2022).

21. Martin, E. W., Holehouse, A. S., Peran, I., Farag, M., Incicco, J. J., Bremer, A., Grace, C. R., Soranno, A., Pappu, R. V. & Mittag, T. Valence and patterning of aromatic residues determine the phase behavior of prion-like domains. *Science* **367,** 694–699 (2020).

22. Janson, G., Valdes-Garcia, G., Heo, L. & Feig, M. Direct generation of protein conformational ensembles via machine learning. *Nat. Commun.* **14,** 774 (2023).

23. Zheng, W., Dignon, G., Brown, M., Kim, Y. C. & Mittal, J. Hydropathy Patterning Complements Charge Patterning to Describe Conformational Preferences of Disordered Proteins. *J. Phys. Chem. Lett.* **11,** 3408–3415 (2020).

24. Hein, M. Y., Hubner, N. C., Poser, I., Cox, J., Nagaraj, N., Toyoda, Y., Gak, I. A., Weisswange, I., Mansfeld, J., Buchholz, F., Hyman, A. A. & Mann, M. A human interactome in three quantitative dimensions organized by stoichiometries and abundances. *Cell* **163,** 712–723 (2015).

25. Ginell, G. M., Flynn, A. J. & Holehouse, A. S. SHEPHARD: a modular and extensible software architecture for analyzing and annotating large protein datasets. *Bioinformatics* **39,** (2023).
