# Supplementary material for: Direct prediction of intrinsically disordered protein conformational properties from sequence
Source: Nat Methods. 2024 Jan 31;21(3):465–76. doi: 10.1038/s41592-023-02159-5 (PMC10927563; doi:10.1038/s41592-023-02159-5)
Supplement: Supplementary file 2 — Reporting Summary [file 41592_2023_2159_MOESM2_ESM.pdf]

nature portfolio

Corresponding author(s): Alex Holehouse
Last updated by author(s): Nov 25, 2023

Reporting Summary

Nature Portfolio wishes to improve the reproducibility of the work that we publish. This form provides structure for consistency and transparency in reporting. For further information on Nature Portfolio policies, see our Editorial Policies and the Editorial Policy Checklist.

Statistics

For all statistical analyses, confirm that the following items are present in the figure legend, table legend, main text, or Methods section.

n/a	Confirmed	
☐	☒	The exact sample size (*n*) for each experimental group/condition, given as a discrete number and unit of measurement
☒	☐	A statement on whether measurements were taken from distinct samples or whether the same sample was measured repeatedly
☐	☒	The statistical test(s) used AND whether they are one- or two-sided<br>*Only common tests should be described solely by name; describe more complex techniques in the Methods section.*
☒	☐	A description of all covariates tested
☒	☐	A description of any assumptions or corrections, such as tests of normality and adjustment for multiple comparisons
☐	☒	A full description of the statistical parameters including central tendency (e.g. means) or other basic estimates (e.g. regression coefficient) AND variation (e.g. standard deviation) or associated estimates of uncertainty (e.g. confidence intervals)
☐	☒	For null hypothesis testing, the test statistic (e.g. *F*, *t*, *r*) with confidence intervals, effect sizes, degrees of freedom and *P* value noted<br>*Give P values as exact values whenever suitable.*
☒	☐	For Bayesian analysis, information on the choice of priors and Markov chain Monte Carlo settings
☒	☐	For hierarchical and complex designs, identification of the appropriate level for tests and full reporting of outcomes
☒	☐	Estimates of effect sizes (e.g. Cohen's *d*, Pearson's *r*), indicating how they were calculated

Our web collection on statistics for biologists contains articles on many of the points above.

Software and code

Policy information about availability of computer code

Data collection	All data in this project was generated using open source software, and all tools are available free of charge. Natural biological amino acid sequences were obtained from UniProt and both those sequences and the associated UniProt IDs are provided. All synthetic sequences were generated using GOOSE, and those synthetic sequences are provided. Yeast sequences used in evolutionary analysis are provided. All data associated with the study are presented at one of:<br>https://github.com/holehouse-lab/supportingdata/tree/master/2023/ALBATROSS_2023<br>and/or doi:10.5281/zenodo.10198621
Data analysis	Software used here included:<br>metapredict V2-FF (https://github.com/idptools/metapredict) version 2.6<br>parrot (https://github.com/idptools/parrot) version 1.7.5<br>sparrow (https://github.com/idptools/sparrow) version 0.2.1<br>soursop (https://soursop.readthedocs.io/) version 0.2.4<br>shephard (https://github.com/holehouse-lab/shephard) version 0.1.19<br>AFRC (https://github.com/idptools/afrc) version 0.3.4<br>LAMMPS (https://www.lammps.org/#gsc.tab=0) version 29 Sep 2021 - Update 2<br>ClustalOmega (http://www.clustal.org/omega/) version 1.2.3<br>CD-HIT (https://github.com/weizhongli/cdhit) version 4.8.1<br>pyMSA (https://github.com/benhid/pyMSA) v0.5.1<br>CAMPARI (https://campari.sourceforge.net/) V2

All notebooks for figures generated in this manuscript are available at https://github.com/holehouse-lab/supportingdata/tree/master/2023/ALBATROSS_2023 (if any are missing PLEASE reach out!)

For manuscripts utilizing custom algorithms or software that are central to the research but not yet described in published literature, software must be made available to editors and reviewers. We strongly encourage code deposition in a community repository (e.g. GitHub). See the Nature Portfolio guidelines for submitting code & software for further information.

Data

Policy information about availability of data

All manuscripts must include a data availability statement. This statement should provide the following information, where applicable:

- Accession codes, unique identifiers, or web links for publicly available datasets
- A description of any restrictions on data availability
- For clinical datasets or third party data, please ensure that the statement adheres to our policy

The only databases used in this study are UniProt (https://uniprot.org/) which is referenced appropriately. Beyond this, all data, code, and analysis used for this manuscript are shared at: https://github.com/holehouse-lab/supportingdata/tree/master/2023/ALBATROSS_2023. Synthetic and natural IDRs used for training and test data are shared in the main GitHub repo, and are also shared as a Zenodo repository (10.5281/zenodo.10198620). Sequences for which small-angle X-ray scattering data and alternative predictive tools were tested are shared in the main GitHub repository. All of the data associated with the proteome-wide analysis presented in Fig. 4 and Fig. 5 are shared as SHEPHARD-compliant datafiles, and we encourage other groups to explore these predictions in the context of other protein annotations using SHEPHARD and the set of precomputed annotations provided therein. All data associated with Fig. 6 are provided as files. In addition all other data and code used for sequence analysis, training weights, bioinformatic data, the SPARROW implementation, and the Google Colab notebook are linked from this manuscript's main GitHub directory.

Human research participants

Policy information about studies involving human research participants and Sex and Gender in Research.

Reporting on sex and gender	N/A
Population characteristics	N/A
Recruitment	N/A
Ethics oversight	N/A

Note that full information on the approval of the study protocol must also be provided in the manuscript.

Field-specific reporting

Please select the one below that is the best fit for your research. If you are not sure, read the appropriate sections before making your selection.

☒ Life sciences ☐ Behavioural & social sciences ☐ Ecological, evolutionary & environmental sciences

For a reference copy of the document with all sections, see nature.com/documents/nr-reporting-summary-flat.pdf

Life sciences study design

All studies must disclose on these points even when the disclosure is negative.

Sample size	Sample sizes were chosen either based on data availability (i.e. we focused on twenty yeast species as homologous proteins were already identified via synteny, the human proteome defines a fixed number of predicted IDRs), based on prior biophysical work (a window size of 51 residues was chosen to match prior work showing ~50 residues enables complex sequence-encoded biophysical behavior to emerge, 100 randomly sequences were randomly generated per sequence composition in Figure 3, as we found trends were consistent and smooth with 40-50 sequences and so doubled this number to ensure were were in a robust regime) or based on the consequences of biophysical space (by systematically varying sequence features we generated 23,127 synthetic sequences, so chose around the same number of biological sequences (19,075) as a starting point.
Data exclusions	No data were excluded.
Replication	Every step in the manuscript can be and was repeated. Simulation error is almost zero, training with the final hyperparameters reliable generates models with equivalent accuracy, and once trained those models are deterministic such that all results in figure 3-6 can be re-generated using the notebooks associated with the GitHub link.
Randomization	When selecting a subset of biological sequences, we randomly selected IDRs from across a set of eukaryotic proteomes based on a length criterion only. This dataset was designed to explicitly sample 'relevant' biological sequence space, such that random sampling offers a means to take advantage of the fact that sequence features more commonly seen in natural proteins will - by definition - by aquired in this random

	sampling than if we tried to systematically explore sequence space. Random sampling here was done simply by uniform probability sampling over the set(s) of IDR sequences that were appropriately lengthed.
Blinding	Blinding was not necessary in this study because there is no point where blinding any aspect would have been helpful/relevant/appropriate.

Reporting for specific materials, systems and methods

We require information from authors about some types of materials, experimental systems and methods used in many studies. Here, indicate whether each material, system or method listed is relevant to your study. If you are not sure if a list item applies to your research, read the appropriate section before selecting a response.

Materials & experimental systems

n/a	Involved in the study
☒	☐ Antibodies
☒	☐ Eukaryotic cell lines
☒	☐ Palaeontology and archaeology
☒	☐ Animals and other organisms
☒	☐ Clinical data
☒	☐ Dual use research of concern

Methods

n/a	Involved in the study
☒	☐ ChIP-seq
☒	☐ Flow cytometry
☒	☐ MRI-based neuroimaging
